# Formation of an RNA Quadruplex-Duplex Hybrid in Living Cells between mRNA of the Epidermal Growth Factor Receptor (EGFR) and a G-Rich Antisense Oligoribonucleotide

**DOI:** 10.3390/cells9112375

**Published:** 2020-10-29

**Authors:** Dorota Gudanis, Damian Kaniowski, Katarzyna Kulik, Daniel Baranowski, Zofia Gdaniec, Barbara Nawrot

**Affiliations:** 1 Institute of Bioorganic Chemistry, Polish Academy of Sciences, 61-704 Poznan, Poland; daniel.baranowski@ibch.poznan.pl (D.B.); zgdan@ibch.poznan.pl (Z.G.); 2 Centre of Molecular and Macromolecular Studies, Polish Academy of Sciences, 90-363 Lodz, Poland; dkanio@cbmm.lodz.pl (D.K.); kpieta@cbmm.lodz.pl (K.K.); bnawrot@cbmm.lodz.pl (B.N.)

**Keywords:** G-quadruplex, quadruplex-duplex hybrid, RNA G-rich antisense oligonucleotide, EGFR mRNA, selective G4 fluorescent probe, oBMVC derivative, distant G-tracts

## Abstract

Antisense DNA oligonucleotides, short interfering RNAs (siRNAs), and CRISPR/Cas9 genetic tools are the most useful therapeutic nucleic acids regulating gene expression based on the antisense specificity towards messenger RNA. Here, we present an effective novel strategy for inhibiting translation based on the antisense-controlled formation of an RNA quadruplex-duplex hybrid (QDH) between a G-rich RNA antisense oligoribonucleotide (Q-ASO) and specific mRNA, comprising two distant G-tracts. We selected epidermal growth factor receptor (EGFR) as a well-established target protein in anticancer therapy. The chemically modified, bi-functional anti-EGFR Q-ASO and a 56-nt long EGFR mRNA fragment, in the presence of potassium ions, were shown to form in vitro very stable parallel G-quadruplex containing a 28-nt long external loop folding to two duplex-stem structure. Besides, the Q-ASOs effectively reduced EGFR mRNA levels compared to the non-modified RNA and DNA antisense oligonucleotides (rASO, dASO). In addition, the hybridization specificity of Q-ASO comprising a covalently attached fluorescent tag was confirmed in living cells by visualization of the G4 green fluorescent species in the presence of other antisense inhibitors under competitive conditions. The results presented here offer novel insights into the potential application of Q-ASOs for the detection and/or alteration of (patho)biological processes through RNA:RNA quadruplex-duplex formation in cellular systems.

## 1. Introduction

G-quadruplexes (G4s) are DNA or RNA structures formed from stacked guanine tetrads. Computational studies have revealed the prevalence of more than 370,000 candidate putative G-quadruplex DNA sequences (PQS) in various regions of the human genome [1]. The initial algorithm was designed to search for all stable G-quadruplexes with loop lengths up to 7 nt. However, the possibility of the formation of G4 structures possessing longer loops (L > 7–20) has been recently demonstrated [2,3,4,5]. The intramolecular base pairing in a long loop within the PQS was shown to be a driving force for the formation of stable quadruplex-duplex structures. The identification of over 80,000 stem loop-containing G-quadruplex sequences (SLQS) in the human genome suggests potential biological roles of these motifs. Interestingly, 48,508 SLQS are located across genic and gene promoter regions. SLQS were also identified in 2429 mature mRNAs, 921 of which were located at 5′-UTRs [5]. Using recently developed approaches, such as deep sequencing methods (NGS), a significantly higher number of G-quadruplexes was identified, highlighting that DNA and RNA sequences eagerly form unconventional structures containing two-layer G-tetrads, bulges, or longer loops [6,7]. The use of fluorescent probes and antibodies demonstrated that the G4 motif is widely distributed throughout the human genome and transcriptome *in vivo* [8,9,10]. Moreover, G4 has been shown to be implicated in key biological processes, including recombination, replication, transcription, and translation [11,12,13]. A connection between G-quadruplex formation and key biological processes in cells can be exploited to design therapeutic and diagnostic tools for a wide range of human diseases [14,15,16,17]. For example, endogenous G-quadruplexes have proven to be attractive targets for cancer therapy. One way to downregulate expression of a pathogenic gene is trapping of the G-quadruplexes in cells by G-quadruplex-specific ligands [18,19,20]. The G4-ligand approach might be particularly relevant for G-quadruplex-dependent interventions in biological processes because they can extend the lifetime of G-quadruplex structures in cells [21]. However, due to low selectivity of the ligands against particular G-quadruplex topologies, undesirable *off*-target effects can occur. The use of short G-rich oligonucleotides (5′-GGGCCCGGG-3′) to form intermolecular G-quadruplex structures with G-rich target sequences is one of the methods used to regulate cellular processes burdened by low recognition specificity [22]. Attachment of a G-rich sequence to a traditional antisense oligonucleotide is another method to improve selective targeting of the G-rich sequence. The use of DNA G-rich antisense oligonucleotide (Q-ASO) with a propensity to form a DNA:RNA quadruplex-duplex hybrid structure relies on the ability of duplex segments (ASO) to hybridize with mRNA in a sequence-controllable fashion. The efficacy of such bifunctional DNA Q-ASOs has been demonstrated in studies concerning the inhibition of reverse transcription in various model RNAs or translation of eIF-4E mRNA [23,24]. The authors tested several variants of bifunctional DNA Q-ASOs for the efficacy of this tool [24]. However, studies concerning the influence of chemical modifications on the functionality of these therapeutic oligonucleotides are limited to phosphorothioate modifications only [23].

Epidermal growth factor receptor (EGFR) is produced by epithelial cells and is the most important growth regulator in these cells. Mutations and overexpression of the EGFR protein are common and occur in many cancer types, such as lung, ovarian, stomach, and head and neck cancers [25]. Several anti-EGFR therapies, such as monoclonal antibodies, tyrosine kinase inhibitors, CRISPR-Cas9, and antisense oligonucleotides, have been developed [26,27,28,29,30]. We previously reported the use of antisense oligonucleotides modified with boron clusters to study the silencing activity toward the EGFR gene [28,30,31].

Here, we present an approach based on the use of chemically modified, bifunctional G-rich oligoribonucleotides, Q-ASOs, to control the formation of RNA quadruplex-duplex hybrid structures on mRNA templates. The formation of this secondary structure inhibits translation by affecting ribosome function. This approach combines the high specificity of an ASO “guide” RNA fragment, with the high efficiency of G-rich sequences (Q) for forming a G-quadruplex with target mRNA. Combining Nuclear Magnetic Resonance (NMR), Ultraviolet-Visible (UV), and Circular Dichroism (CD) spectroscopic techniques and gel electrophoresis we demonstrated, for the first time, the formation of bimolecular, RNA:RNA quadruplex-duplex hybrid structures (QDH), containing a 28-nt external loop (forming a two duplex stem structure, 2ST). The biological stability of these structures was enhanced by the introduction of non-naturally occurring chemical modifications, such as non-nucleotide linkers or fluorinated analogs of guanosine.

Our experimental data reveal that incorporation of G-rich segments into RNA ASOs results in significantly higher activity of Q-ASOs relative to conventional rASOs or dASOs (Table 1). Their low toxicity, sequence specificity, and high silencing efficiency make them potential candidates for anticancer therapy. Importantly, the possibility of targeting mRNA comprising two distant G-tracts is demonstrated for the first time. In all previously described quadruplex-duplex antisense approaches, the distance between G-tracts in the target molecule was not greater than 3-nt [23,24].

## 2. Materials and Methods

All RNA and DNA sequences used in the study are shown in Table 1.

### 2.1. Synthesis and Purification of Unmodified Short DNA and RNA Oligonucleotides

Synthesis of DNA and RNA oligonucleotides at the 0.1 µmole scale was performed according to routine procedures on a Gene World DNA synthesizer (K&A, Schaafhein, Germany) under the conditions recommended by the manufacturer. All compounds were cleaved from the solid support, deprotected using standard procedures, and then purified by RP-HPLC. The molecular mass of all synthesized oligonucleotides was confirmed by ESI-Q-TOF mass spectrometry (Waters Corp., Milford, MA, USA). The purity of compounds was analyzed by 20% polyacrylamide gel electrophoresis (PAGE) and RP-HPLC performed on a Shimadzu Prominence HPLC system (Shimadzu Corp., Kyoto, Japan) using a Kinetex 5 µm, C18, 250 × 4.6 mm column with buffer A (0.1 M CH_3_COONH_4_/H_2_O) and buffer B (100% CH_3_CN) at a 1 mL/min flow rate. The buffer gradient was as follows: (1) 0–2 min 0% B; (2) 2–21 min 0–40% B; (3) 21–24 min 40–45% B; (4) 24–28 min 45–0% B; and (5) 28–32 min 0% B. UV detection was performed at λ_max_ 268 nm. RP-HPLC profiles and mass spectra of dASO, rASO, and Q-RNA (Table 1) are shown in Figures S10–S12.

To mimic the physiological ionic strength and to prevent dimerization of the G-quadruplex units, spectra were recorded at 50 mM KCl and 100 mM LiCl (Li^+^ does not promote G4 formation) [32].

### 2.2. Synthesis and Purification of Modified Q-ASO and EGFR mRNA Fragment

The long oligonucleotides, the abasic/(CH_2_)_6_-linker-containing RNA (QL-ASO), and 5′-AGCAGCGCCAGGAGCG-AAA-GGGG-UC-GGGGA-linker-NH_2_ RNA (Q-ASO-NH_2_) for preparation of Fl-Q-ASO, were commercially synthesized (ChemGenes Corporation, Wilmington, MA, USA) on the 1 μmol scale. The synthesis of 2′-FANA-G-containing RNA (QF-ASO), as well as the EGFR mRNA fragment (56-nt oligonucleotide), was performed in house using phosphoramidite chemistry on the solid support on the 1 μmol scale on a BioAutomatic MerMade 12 DNA/RNA synthesizer (BioAutomation, Irving, Texas, USA). After automated synthesis, QF-ASO and EGFR oligonucleotides were detached from the support and deprotected according to the manufacturer’s protocol [Glen Research, Glen-Pak RNA Cartridge Purification (DMT-ON), Sterling, VA, USA]. QF-ASO, QL-ASO, Q-ASO-NH_2_, and EGFR oligonucleotides were desalted using Amicon^®^ Ultra 3K centrifugal filters (Merck, Millipore, Darmstadt, Germany) by loading on the filter, washing with 4000 µL MilliQ water and then washing successively against ∼150 mM LiCl and against water. The purity and homogeneity of Q-ASO and EGFR mRNA oligomers were verified by 15% denaturing gel electrophoresis (Figure S13).

### 2.3. Q-ASO-NH_2_ Labeling with oBMVC-C3

The Q-ASO-NH_2_ oligonucleotide 5′-AGCAGCGCCAGGAGCGAAAGGGGUCGGGGA-linker-NH_2_-3′) dissolved in sodium phosphate buffer (0.1 M, pH 7.0) was combined with 5-fold molar excess of in house made oBMVC-C3-NHS ester in DMSO (Figure S7). The mixture was incubated at 37 °C in the dark overnight. The oligo-oBMVC-C3 conjugate (Fl-Q-ASO) (Figure 8) was separated from the salts and free oBMVC-C3-NHS by 2% NaClO_4_/acetone precipitation. The purity and homogeneity of the oligo-oBMVC-C3 conjugates were verified by 15% denaturing gel electrophoresis (Figure S13).

### 2.4. Denaturing Electrophoresis of RNA Oligonucleotides

First, 350 pmole RNAs (QL-ASO, EGFR) or (QF-ASO, QL-ASO, Fl-Q-ASO) were suspended in 4 µL water and 4 µL of 8 M urea (in 8 μL final volume). Next, samples were heated at 95 °C for 3 min, cooled to room temperature, and loaded on 15% TBE-Urea gels (Invitrogen, Termo Fisher Scientific, Pittsburgh, PA, USA). Denaturing gel electrophoresis experiments were performed in 1.5× TBE buffer. Electrophoresis experiments were run at 180 V for 2 h and gels were viewed by UV shadowing (Figure S13).

### 2.5. Non-Denaturing Electrophoresis of RNA Oligonucleotides

First, 350 pmole of RNAs were suspended in 10 mM Tris-HCl, pH 6.8, with 150 mM LiCl and 50 mM KCl (in 6 μL final volume) (Figure 2) or in addition with two equivalents of NMM (in 6 μL final volume) (Figure S4). Next, samples were heated at 95 °C for 3 min and gradually cooled to room temperature (temperature decrease of 1 °C/min). Subsequently, they were mixed with 2 μL of 50% glycerol and loaded on 20% TBE gels (Invitrogen, Termo Fisher Scientific, Pittsburgh, Pennsylvania, USA). O’RangeRuler 5 bp and DNA ladder containing 50 and 100 bp as brighter bands (Thermo Fisher Scientific, Pittsburgh, PA, USA) were used as molecular markers. Gel electrophoresis experiments performed in a 0.5 × TBE buffer were run at 180 V for 2.5 h at 4 °C (an ice bath), and the first gel was viewed by UV shadow (image, Figure 2A) and *N*-methyl mesoporphyrin IX staining (NMM; Frontier Scientific, Newark, DE, USA) as previously described (Figure 2B) [33]. After post-staining the gel with NMM, the gel was scanned with a Fuji FLA-5100 imaging system (Fujifilm Life Sciences, Cambridge, MA, USA) (Figure 2B). The second gel was viewed by UV shadow (image, Figure S4A) and to visualize RNA adopting G-quadruplex structures in the presence of K^+^/NMM (Figure S4B) or fluorescently labeled G-quadruplex (Fl-Q-ASO/EGFR) (Figure S8), the gels were only exposed to 573 nm light.

### 2.6. NMR Experiments

NMR experiments were performed on a 700 MHz Bruker AVANCE III spectrometer (Bruker Corporation, Billerica, MA, USA) equipped with a QCI CryoProbe. The ^1^H NMR spectra were recorded at 25 °C, and 40 °C in 3 mm thin wall tubes with water suppression using an excitation sculpting scheme with gradients, typically from 2000–3000 scans with 1.0 s relaxation delays. RNAs (~0.055 mM) were dissolved in the final sample volume of 0.2 mL (90% H_2_O/10% D_2_O) in 150 mM LiCl, 10 mM Tris-HCl, pH 6.8 or to the samples indicated below the drawing were then added to 10.5 µL 3 M KCl. For each sample, the spectrum was recorded 10 min after annealing.

### 2.7. UV Thermal Denaturation Curves

First, 560 pmole of QF-ASO/EGFR and QL-ASO/EGFR complexes were dissolved in a buffer containing 50 mM KCl, 150 mM LiCl, and 10 mM Tris-HCl at pH 6.8. Thermal denaturation curves were obtained by monitoring at 260 and 295 nm with a JASCO V-650 spectrophotometer (JASCO International Co., Ltd., Tokyo, Japan) using quartz optical cuvettes of 0.5 path lengths with the sample volume (150 µL). Samples were protected against evaporation by silicone oil. The temperature range was 20–90 °C, using a scan rate of 0.5 °C min^−1^. Spectra were processed and prepared using Origin 8 Software (OriginLab Corporation, Northampton, MA, USA).

### 2.8. CD Measurements

CD spectra were measured using a Jasco model J-815 CD spectrophotometer (JASCO International Co., Ltd., Tokyo, Japan) over the range of 220–330 nm using a 0.5 cm path length quartz cuvette with a reaction volume of 750 μL. Solutions for CD spectra were prepared by taking 0.14 mL samples after UV experiment and diluting to 0.75 mL with 50 mM KCl solution. The final concentrations of QF-ASO/EGFR and QL-ASO/EGFR were ~0.8 µM. Spectra were processed and prepared using Origin 8 Software.

### 2.9. Cell Lines and Culture Conditions, Dual Fluorescence Assay

HeLa (human cervical carcinoma, ATCC, Manassas, Virginia, USA) cells were cultured in RPMI 1640 medium (Gibco, BRL, Paisley, New York, NY, USA) supplemented with 10% heat-inactivated fetal bovine serum (FBS) (Gibco, BRL, Paisley, New York, NY, USA), 100 U/mL penicillin, and 100 μg/mL streptomycin (Gibco, BRL, Paisley, New York, NY, USA) at 37 °C and 5% CO_2_. A431 (human squamous carcinoma), MCF-7 (human breast cancer) cell lines (ATCC, Manassas, Virginia, USA) were cultured in DMEM medium containing 4.5 g/L D-Glucose, 0.11 g/L sodium pyruvate and without L-glutamine (Gibco, BRL, Paisley, New York, NY, USA) supplemented with 10% heat-inactivated fetal bovine serum (Gibco, BRL, Paisley, New York, NY, USA), 100 U/mL penicillin, and 100 µg/mL streptomycin (Gibco, BRL, Paisley, New York, NY, USA) at 37 °C and 5% CO_2_. Right before transfection, the culture medium was replaced, and cells were trypsinized and counted. Twenty four hours before the experiment, cells were plated in 96-well plate (plates with black walls and transparent bottom, Perkin–Elmer, Waltham, MA, USA) at a density of 15 × 10^3^ cells per well in a volume of 100 µL full medium. On the day of transfection, the cell culture should be 80% confluent. Directly before transfection, the cell medium containing antibiotics was replaced with the new medium free of antibiotics at 100 µL per well.

Transfections were performed using the Lipofectamine 2000 transfection reagent (Invitrogen, New York, NY, USA) at a ratio of 2:1 (2 μL of Lipofectamine 2000 per 1 μg of nucleic acid (d-ASO-C, dASO, rASO, Q-RNA, QF-ASO, QL-ASO) according to the manufacturer’s protocol). For dual fluorescence assay (DFA), HeLa cells were transfected with DNA plasmids: reporter plasmid pDsRed-N1 (BD Biosciences) (45 ng/well) and pEGFP-EGFR plasmid [28,30] (100 ng/well) and suitable oligonucleotides) (dASO-C, dASO, rASO, Q-RNA, QF-ASO, QL-ASO) (50–200 nM) dissolved in OPTI-MEM medium in 50 µL (GIBCO, BRL, Paisley, New York, NY, USA). After 5 h of incubation, the transfection mixture was replaced with 200 µL fresh medium per well containing antibiotics. After 48 h of incubation at 37 °C in a 5% atmosphere of CO_2_, cells were washed twice with PBS buffer (without Ca^2+^ and Mg^2+^) and lysed in NP-40 buffer (150 mM NaCl, 1% IGEPAL, 50 mM Tris-HCl pH 7.0, 1 mM PMSF) overnight at 37 °C. Prepared cell lysates were used for fluorescence measurements.

Fluorescence values of pEGFP-EGFR (green fluorescent protein) and RFP (red fluorescent protein) were determined using a Synergy HT reader (BIO-TEK). Quantification of data was performed using KC4 software. Excitation and emission wavelengths for each protein were as follows: EGFP − λ_Ex_ = 485/20 nm and λ_Em_ = 528/20 nm and RFP: λ_Ex_ = 530/25 nm and λ_Em_ = 590/30 nm. Oligonucleotide activity was calculated as the ratio of EGFP to RFP fluorescence values according to the following equation: activity of oligonucleotide (%) = 100% − (sample EGFP-X/RFP: control EGFP-X/RFP) × 100%, where EGFP-X indicates the fluorescence value of the EGFP-X fusion protein (X = EGFP-EGFR). Average fluorescence values were the means of eight repeats calculated after eliminating extreme values. Each oligonucleotide activity value given on the plots is the average of mean values from three independent experiments. The level of fluorescence (EGFP-EGFR/RFP) in control cells (transfected with pDsRed-N1 and pEGFP-EGFR plasmids) was taken as a reference (100%).

### 2.10. Cytotoxicity of Antisense Oligomers dASO-C, dASO, rASO, Q-RNA, QF-ASO, QL-ASO in HeLa Cells

The cytotoxicity of oligonucleotides (dASO-C, dASO, rASO, Q-RNA, QF-ASO, QL-ASO) in HeLa cells was assessed via the MTT assay. Cells were plated at a density of 8,000 cells per well in 96-well plates. Cells were treated with ASO (50–200 nM), and the control was considered as having 100% viability. Cells were incubated for 48 h at 37 °C in 5% CO_2_, followed by the addition of MTT solution in PBS (5 mg/mL) to each well. Cells were then incubated for 3 h at 37 °C in 5% CO_2_. Finally, 95 μL lysis buffer (NP-40, 20% SDS, 50% aqueous dimethylformamide, pH 4.5) was added to each well and incubated overnight at 37 °C. Absorbance was measured at two wavelengths, 570 nm and the reference wavelength of 630 nm (colorless walls plate reader, Perkin-Elmer). Cell viability (cell mitochondrial activity) was determined as previously described [28].

### 2.11. Microscopic Analysis of Fluorescent Cells Expressing EGFP-EGFR Protein from the pEGFP-EGFR Fusion Plasmid

HeLa cells were transfected as described above with the pDsRed-N1 reporter plasmid (45 ng/well) and pEGFP-EGFR fusion plasmid (100 ng/well), as well as the antisense oligonucleotide QL-ASO (Table 1) at concentrations of 50, 100, and 200 nM dissolved in 50 µL OPTI-MEM medium. Cells transfected with the pDsRed-N1 and pEGFP-EGFR plasmids were used as a reference (100%). For the dual fluorescence assay, after 5 h of incubation, the transfection mixture was replaced with fresh medium containing antibiotics in 200 µL per well. After 48 h of incubation at 37 °C in an atmosphere of 5% CO_2_, cell medium was replaced with PBS buffer (with Ca^2+^ and Mg^2+^), and the cells were imaged under a fluorescence microscope (Nikon-Eclipse, Japan) with FITC (λ_ex_ = 465–495, λ_DM_ = 505, λ_BA_ = 515–555), B-2A (long-pass, λ_ex_ = 450–490, λ_DM_ = 505, λ_BA_ = 520), TX red (ex λ_ex_ = 540–580, λ_DM_ =595, λ_BA_ = 600–660), and G-2A (long-pass λ_ex_= 510–560, λ_DM_ = 575, λ_BA_ = 590), where DM is a dichroic mirror and BA is an absorption filter.

### 2.12. Microscopic Analysis of Localization of Quadruplexes Formed by Fl-Q-ASO (100 nM) and EGFR mRNA in MCF-7, HeLa and A431 Cells

MCF-7 (human breast cancer) and A431 (human squamous carcinoma) cell line were cultured in DMEM medium containing 4.5 g/L D-Glucose, 0.11 g/L Sodium Pyruvate and without L-Glutamine (Gibco, BRL, Paisley, New York, NY, USA) and HeLa cell line were cultured in RPMI 1640 medium (Gibco, BRL, Paisley, New York, NY, USA). Medium was supplemented with 10% heat-inactivated fetal bovine serum (FBS) (Gibco, BRL, Paisley, New York, NY, USA), 100 U/mL penicillin, and 100 μg/mL streptomycin (Gibco, BRL, Paisley, New York, NY, USA) at 37 °C and 5% CO_2_.

Right before transfection, the culture medium was replaced, the cells were trypsinized and counted. Twenty-four hours before the experiment, cells were plated in 96-well plate (plates with black walls and transparent bottom, Perkin-Elmer, Waltham, MA, USA) at the density of 15 × 10^3^ cells per well in a volume of 100 µL of full medium. On the day of transfection, the cell culture should be 80% confluence. Directly before the transfection, the cell medium containing antibiotics was replaced with the new one, free of antibiotics in a volume of 100 µL per well.

Transfection was executed using Lipofectamine 2000 transfection reagent (Invitrogen, New York, NY, USA) at a ratio 2:1 (2 μL of Lipofectamine 2000 per 1 μg of the nucleic acid (FL-Q-ASO, 100 nM) according to the manufacturer’s protocol. After 5 h of incubation, the transfection mixture was replaced with fresh medium with antibiotics in a volume of 200 µL per well and mixed with 5 μg/mL DAPI dye (stained in blue cell nucleic) (Invitrogen, ThermoFisher Scientific, Massachusetts, USA) and 1 μM/mL ER-Tracker Red dye (stained in red ER membranes cell) (Invitrogen, ThermoFisher Scientific, Massachusetts, USA). The cells were incubated for 30 min with dyes protected from light at 37 °C. After incubation the cells were washed two times with PBS buffer (with Ca^2+^ and Mg^2+^) and the cells were imaged under a fluorescence microscope (Nikon-Eclipse, Japan) with DAPI (λ_ex_ = 340–380, λ_DM_ = 400, λ_BA_ = 435–485) and FITC (λ_ex_ = 465–495, λ_DM_ = 505, λ_BA_ = 515–555), B-2A (long-pass, λ_ex_ = 450–490, λ_DM_ = 505, λ_BA_ = 520) and TX red (ex λ_ex_ = 540–580, λ_DM_ = 595, λ_BA_ = 600–660), G-2A (long-pass λ_ex_ = 510–560, λ_DM_ = 575, λ_BA_ = 590), where DM is a dichroic mirror and BA is an absorption filter.

### 2.13. Microscopic Analysis of Hybridization of Fl-Q-ASO with Endogenous EGFR mRNA in A431 Cells in the Presence of Various Inhibitory ASOs

A431 cells were cultured as described above. Transfection was executed using Lipofectamine 2000 transfection reagent (Invitrogen, New York, NY, USA) at a ratio 2:1 (2 μL of Lipofectamine 2000 per 1 μg of the nucleic acid) with Fl-Q-ASO (100 nM) and the following inhibitory ASOs (100 nM): rASO, QRNA, rASO+QRNA (1:1 molar ratio), QL-ASO and dASO-R.

After 5 h of incubation, the transfection mixture was replaced with fresh medium with antibiotics in a volume of 200 µL per well. After next 48 h of incubation at 37 °C in 5% atmosphere of CO_2_, the cells were washed two times with PBS buffer (with Ca^2+^ and Mg^2+^), and imaged under a fluorescence microscope (Nikon-Eclipse, Japan) with DAPI (λ_ex_ = 340–380, λ_DM_ = 400, λ_BA_ = 435–485) and FITC (λ_ex_ = 465–495, λ_DM_ = 505, λ_BA_ = 515–555), B-2A (long-pass, λ_ex_ = 450–490, λ_DM_ = 505, λ_BA_ = 520), where DM is a dichroic mirror and BA is an absorption filter.

## 3. Results

### 3.1. Hybridization of Q-ASO and EGFR mRNA Induces Quadruplex-Duplex Hybrid Formation

**Figure 1:**
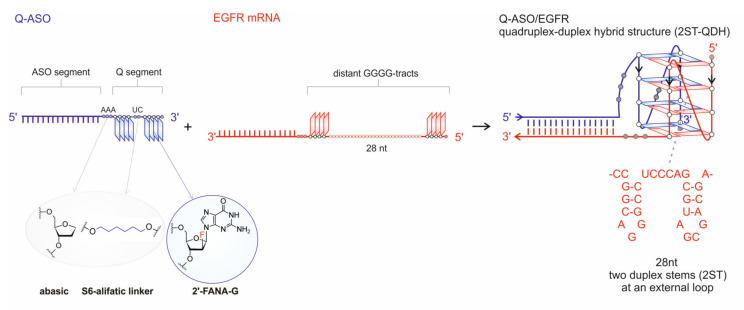
Schematic representation of Q-ASO and the EGFR mRNA target and their hybridization to form a quadruplex-duplex hybrid containing an external loop folded into two duplex stems (2ST-QDH). The sequences and abbreviations of the chemically modified antisense oligonucleotides and EGFR target are provided in Table 1.

The therapeutic strategy proposed in this work (Figure 1) is based on the ability of an EGFR mRNA target comprising two distant GGGG-tracts to fold into a G-quadruplex after hybridization with a Q segment of a Q-ASO oligonucleotide. The second domain of Q-ASO, ASO, is complementary to the 3′-end of target sequence and is responsible for recognizing sense fragment of mRNA adjacent to the G-tract via duplex formation. The 56-nt target corresponds to the 5′-UTR and coding region of the EGFR gene (Figure S1) and contains two GGGG-tracts separated by a 28-nt fragment. This fragment may adopt a two-duplex stem (2ST) stable secondary structure, bringing distant G-tracts close together and promoting formation of the quadruplex-duplex hybrid, as shown in Figure 1. To increase the propensity of target sequences containing two distant G-tracts to fold into a G-quadruplex after hybridization with Q-ASO, we obtained two model oligonucleotides bearing the chemical modifications known to stabilize DNA G-quadruplexes [34,35,36]. Into one of the Q-ASO sequences, abasic residues between the Q and ASO segments were introduced in place of unpaired residues (AAA), while an aliphatic spacer was inserted between the two G-tracts instead of UC residues (Table 1). Furthermore, the guanosine analog (2′-FANA-G) was introduced into the second sequence, instead of the two internal guanosine residues in the G-tracts (Table 1, Figure 1).

### 3.2. Native Polyacrylamide Gel Electrophoresis Validation of Secondary Structures of Q-ASO and EGFR and Their Complexes

**Figure 2:**
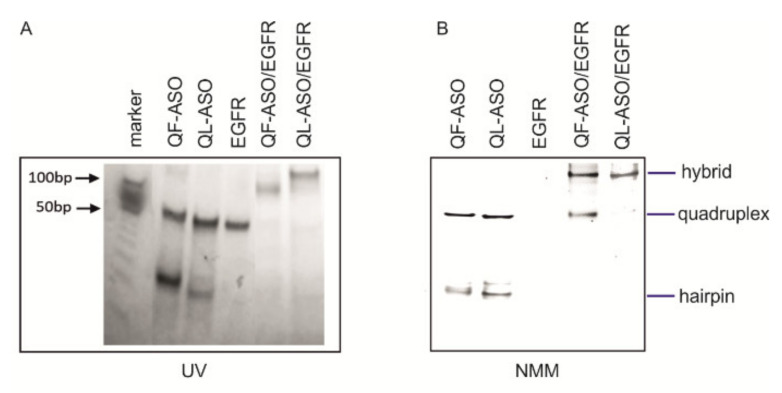
Analysis of QF-ASO, QL-ASO, EGFR, QF-ASO/EGFR and QL-ASO/EGFR structures by non-denaturing PAGE. All oligomers in 0.1 M Tris HCl, pH 6.8, with 50 mM KCl and 150 mM LiCl were annealed and subjected to non-denaturing PAGE. Next, the gels were visualized by UV shadowing (**A**), post-stained with NMM and exposed to 532 nm light (**B**).

To explore the physicochemical properties and structure of Q-ASO/EGFR hybrids, we used a number of experimental methods. Initially, native polyacrylamide gel electrophoresis (PAGE) was performed to assess the structure of the analyzed molecules in the presence of K^+^ ions based on their electrophoretic mobility. The first lane in Figure 2A corresponds to the DNA ladder marker in the range of 10 bp to 100 bp. The additional lanes in Figure 2A and in Figure 2B correspond to QF-ASO, QL-ASO, and EGFR target oligonucleotides and to the QF-ASO/EGFR and QL-ASO/EGFR complexes, respectively. QF-ASO and QL-ASO oligonucleotides appear as two bands characterized by fast and slow electrophoretic mobility. The EGFR target runs as a single band corresponding to ~50 mer of the DNA ladder marker. Using RNAstructure software, this molecule was predicted to fold into the secondary structure shown in Figure S3A. For QF-ASO/EGFR and QL-ASO/EGFR complexes, the appearance of very retarded bands was observed. These bands migrated similarly to the 100 bp DNA ladder marker and may represent bimolecular structures. After post-staining the gel with *N*-methylmesoporphyrin IX (NMM), which interacts selectively with parallel-stranded G-quadruplexes, only some bands were visible. In the case of QF-ASO and QL-ASO, adopting two primary conformations, only the slower migrating conformers were stained with NMM, suggesting the formation of bimolecular G-quadruplexes (Figure S2). Conformations corresponding to faster-migrating bands could be attributed to single strands (ss). For bands assigned to the bimolecular QF-ASO/EGFR and QL-ASO/EGFR structures (Figure 2A, UV shadow), the observed increased fluorescence intensity upon NMM binding *(*Figure 2B) indicated the formation of a hybrid structure containing the G-quadruplex domain.

Next, the experiment was repeated in slightly different conditions (Figure S4). Before loading on a gel, samples were annealed in a buffer also containing NMM ligand. Comparison of these two gels (Figure 2B vs. Figure S4B) shows that the intensity of bands corresponding to QF-ASO/EGFR and QL-ASO/EGFR hybrids is considerably stronger. The NMM ligand promoted the formation of the G-quadruplex domain.

### 3.3. NMR Validation of QF-ASO/EGFR and QL-ASO/EGFR Folding Topology

**Figure 3:**
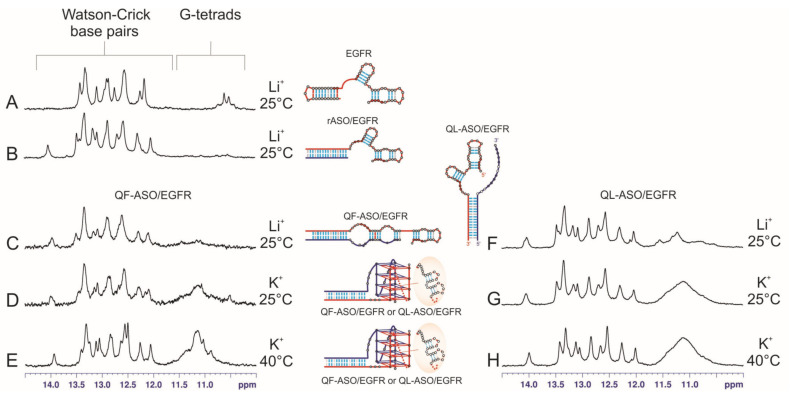
Imino region of the ^1^H NMR spectra of (**A**): EGFR, (**B**): rASO/EGFR, (**C**–**E**, on the left): QF-ASO/EGFR and (**F**–**H**, on the right): QL-ASO/EGFR. Putative secondary structures are shown schematically. All spectra were recorded in 90% H_2_O/10% D_2_O (*v/v*). Symbol Li^+^ corresponds to buffer containing 150 mM LiCl, 10 mM Tris-HCl, 0.1 mM EDTA, pH 6.8; and K^+^ denotes buffer containing 50 mM KCl, 150 mM LiCl, 10 mM Tris-HCl, 0.1 mM EDTA, pH 6.8. Corresponding temperatures are indicated in the figure.

To confirm that both QF-ASO/EGFR and QL-ASO/EGFR adopt quadruplex-duplex hybrid structures, we used NMR spectroscopy. In general, in ^1^H NMR spectra, imino proton peaks associated with Watson–Crick base pairs resonate between 12.0 to 15.0 ppm and those from Hoogsteen base pairs between 10.5 to 12 ppm [37]. The high-field resonances are also characteristic of G-tetrads formed by Hoogsteen hydrogen bonding to guanine residues. In principle, in the imino region of the ^1^H NMR spectrum, the number of observed imino resonances should correspond to the number of stable Watson-Crick G-U base pairs and G-tetrads. Analysis of NMR spectra of larger molecules can be difficult due to the increased line widths associated with slower tumbling, as well as the spectral overlap from the large number of unique signals. Taking this into account, we compared the ^1^H NMR spectra of the different molecules (Figure 3, Figure S3), such as the EGFR target (Figure 3A), complexes of a shorter Q-ASO analog containing only the ASO segment complementary to the 3′-end of EGFR (rASO) with EGFR (rASO/EGFR) (Figure 3B) and complexes obtained after annealing of EGFR with Q-ASOs (QF-ASO/EGFR, QL-ASO/EGFR) (Figure 3C–E,F–H) in lithium or potassium buffer to identify fingerprints of duplex and G-quadruplex domains. According to RNAstructure prediction, EGFR folds into a hairpin structure harboring three duplex stems (Figure S3A). A short rASO oligonucleotide was designed to form a 16-bp duplex with the 3′-end of the EGFR sequence after hybridization (Figure S3B). Secondary structures of QF-ASO/EGFR and QL-ASO/EGFR predicted by RNAstructure are shown in Figure S3C,D, respectively. As shown in Figure S3B–D, these structures all share the same 16-bp duplex fragment (dotted, grey area). This duplex is also expected to form in a quadruplex-duplex hybrid (Figure S3E). Given these findings, we compared the ^1^H NMR spectra of rASO/EGFR, QF-ASO/EGFR, and QL-ASO/EGFR recorded in the presence of Li^+^ cations to identify a pattern characteristic of duplex domains in quadruplex-duplex hybrids (Figure 3B,C,F). It is commonly known that these ions do not promote G-quadruplex formation, however, Li^+^ cations can stabilize duplex or hairpin structures. The spectra of rASO/EGFR, QF-ASO/EGFR, and QL-ASO/EGFR were very similar, likely due to severe crowding in this region that resulted in several expected signals from hairpin fragments not being recognized. Importantly, three spectra differed significantly from that recorded for EGFR in the same conditions (Figure 3A). In the next step, the addition of K^+^ cation induced folding of the G-quadruplex domains of QF-ASO/EGFR and QL-ASO/EGFR (Figure 3D,G). About 10 min after adding K^+^ to the sample, a new, very broad signal appeared in ^1^H NMR spectrum (Figure 3D,G) attributed to imino protons of the G-tetrads. This broad signal sharpened as the temperature increased to 40 °C (Figure 3E,H), supporting the formation of G-quadruplex domains. In buffer containing potassium ions, the imino peak patterns of the Watson–Crick regions did not change, indicating the presence of a 16-bp duplex domain. The NMR spectra shown in Figure 3 indicate that both QF-ASO/EGFR and QL-ASO/EGFR complexes form quadruplex-duplex hybrid structures.

Formation of the QDH structure, containing a four-layer G-quadruplex domain (Figure 1), results in bringing two distant G-tracts of EGFR close together. As shown recently for G-quadruplexes with long loops, formation of a stem-loop guides the G4 folding and accelerates its folding kinetics compared to an unstructured loop counterpart but is not required [38]. We postulate that the 28-nt fragment of the EGFR sequence folds into two duplex stems in place of an external loop of the G-quadruplex domain (Figure 1). It was not possible to directly confirm the structuring of the long loop in Q-ASO/EGFR complexes because the imino protons originating from the two potential duplex-stems (2ST) are expected to resonate in a crowded Watson–Crick region. However, we performed an additional experiment for a molecule corresponding to a 28-nt fragment of the EGFR target. The imino region of the ^1^H NMR spectra recorded at 25 °C and 40 °C for this 28-nt fragment of EGFR are shown in Figure S5. The number of observed imino resonances is in agreement with the secondary structure predicted for this 28-nt fragment (two-stem structure (2ST), Figure S5). Additionally, the presence of all resonances at elevated temperature indicates that this two-stem structure is quite stable.

In summary, the formation of quadruplex-duplex hybrid structures under K^+^-containing conditions was successfully verified by NMR spectroscopy. We further employed UV and CD methods to obtain information on the stability of these structures.

### 3.4. Thermal Stability of 2ST-QDHs

**Figure 4:**
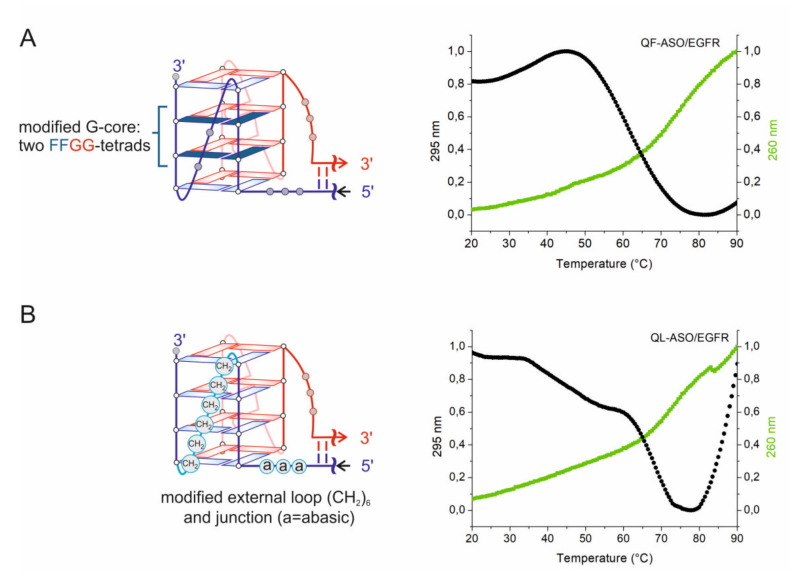
Models and melting curves of quadruplex-duplex hybrids (2ST-QDH). On the panel (**A**), the model with modified 2′-FANA-G marked in dark blue is presented. Panel (**B**) contains second model where non-nucleotide modifications are circled. Melting curves for QF-ASO/EGFR and QL-ASO/EGFR were monitored at 260 nm at 295 nm, in a buffer containing 50 mM KCl, 150 mM LiCl and 10 mM Tris-HCl at pH 6.8.

Applying UV spectroscopy, G-quadruplex melting can be followed by recording absorbance at 295 nm rather than 260 nm, the wavelength used to monitor duplex denaturation. The 295 nm, due to a significant change in absorbance (50–80%), is the preferred wavelength for obtaining melting data of G-quadruplexes [39]. The melting profile of a G-quadruplex at 295 nm is inverted in relation to the profile of the duplex structure. QF-ASO/EGFR and QL-ASO/EGFR hybrids contain in their structures both duplex and G-quadruplex motifs, thus the UV melting curves were recorded at both wavelengths. The melting profiles obtained in the presence of 50 mM potassium ions are presented graphically in Figure 4. The observation of inverted melting profiles obtained at 295 nm for QF-ASO/EGFR and QL-ASO/EGFR hybrids are characteristic of G-quadruplexes and confirmed the formation of G-quadruplex domains. Apparent melting temperatures of QF-ASO/EGFR and QL-ASO/EGFR were determined based on the analysis of curves recorded at 260 nm and 295 nm. T_m_ for QF-ASO/EGFR and QL-ASO/EGFR was ∼74.6 °C and 73.5 °C at 260 nm and 61.3 °C and 67.7 °C at 295 nm, respectively (Figure 4). These results indicate that both quadruplex-duplex hybrids form very stable structures. Comparison of melting temperatures obtained at 295 nm indicates that incorporation of non-nucleotide modifications contributes to the stability of G-quadruplex domains more than the 2′-FANA-G analog.

### 3.5. Topologies of G-Quadruplex Cores of 2ST-QDHs

Due to the unique spectral signatures, CD spectroscopy is widely used to characterize topology of G-quadruplexes. The CD spectrum of antiparallel G-quadruplex displays a negative band at 265 nm and a positive band at 290 nm. The CD spectrum of typical parallel G-quadruplexes exhibits an absorption maximum at 265 nm and minimum at 240 nm. Both G-quadruplex types display an additional positive peak at 210 nm [40]. In contrast, the CD spectrum of A-form RNA is characterized by a dominant positive band at 260 nm and negative bands at 240 nm and 210 nm [41]. As a consequence, analysis of CD spectra of RNA quadruplex-duplex hybrids is complicated, as a spectrum of RNA G-quadruplex adopting principally parallel topology is very similar to that of an A-form RNA duplex. Nevertheless, being aware of this ambiguity, we used CD spectroscopy to confirm that chemical modifications to G-quadruplex domains do not change their topology. Although RNA G-quadruplexes have a high tendency to adopt a parallel topology, exceptions to this rule have been observed [42,43]. In the presence of K^+^ ions, the CD spectra recorded for QF-ASO/EGFR and QL-ASO/EGFR hybrids displayed only a positive band at ~265 nm and small negative band at 240 nm (Figure S6), supporting parallel topology of the G-quadruplex domains.

### 3.6. Silencing Activity of Antisense Oligonucleotides in HeLa Cells Assessed by a Dual Fluorescence Assay

To assess the EGFR silencing activity of the investigated antisense oligonucleotides, we used a dual fluorescence assay (DFA) that was previously developed in our laboratory [28,30,31]. In this assay, HeLa cells are transfected with two plasmids encoding a gene of the target protein (EGFR) fused to the gene for green fluorescence protein (endogenously absent) and a gene for red fluorescence protein (pDsRED-N1, a control gene). The EGFR gene fragment present in the pEGFP-EGFR plasmid (318 nt long, from 227 to 544 nt, mRNA access number NM_005228.4 (NCBI Reference Sequence)) contained a mRNA target sequence (235–290 nt) with two GGGG-tracts and a sense RNA fragment recognized by QF-ASO and QL-ASO antisense RNA oligonucleotides. Both these Q-ASO oligonucleotides contain a so-called “guide” fragment (ASO) and a G-quadruplex forming segment (Q) (Figure 1). Successful transfection of the cells with either of the above listed antisense oligonucleotides (Table 1) should result in decreased expression of the green fluorescence protein, what would be manifested as a reduced green fluorescence signal compared to the control, dASO-C transfected cells, while the expression of red fluorescence protein should remain unchanged [44].

**Figure 5:**
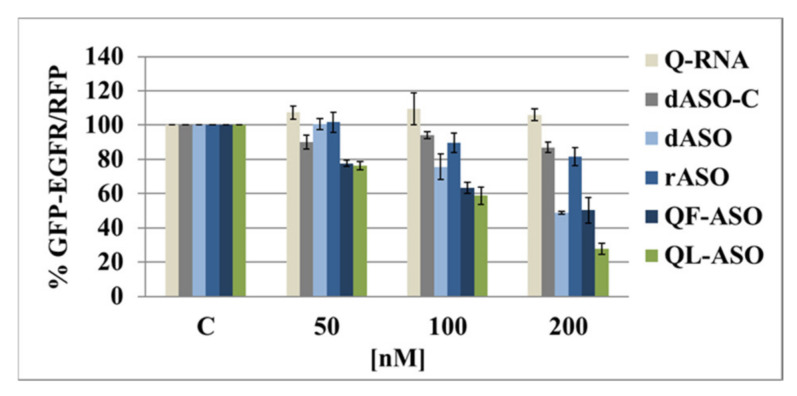
Concentration-dependent silencing activity of dASO, rASO, Q-RNA, QF-ASO and QL-ASO antisense oligonucleotides in HeLa cells determined by the pEGFP-EGFR/RFP dual fluorescence assay. Cells were transfected with pEGFP-EGFR and pDsRED-N1 plasmids and then with the respective antisense oligonucleotides in the concentration range of 50–200 nM using Lipofectamine reagent. Levels of relative EGFP/RFP fluorescence in cells transfected with an empty pEGFP plasmid and reference pDsRED-N1 plasmid were set as 100%. The dASO-C oligonucleotide (100 nM) was used as a control. Results are presented as the mean values ± standard deviation from four independent experiments.

HeLa cells expressing EGFP-EGFR and RFP-coding plasmids were transfected with 50, 100, and 200 nM solutions of antisense oligonucleotides dASO, rASO, Q-RNA, QF-ASO, and QL-ASO (Table 1), and after 48 h incubation, fluorescence was measured with a plate reader. As shown in Figure 5, two short oligomers dASO and rASO, complementary to the sense fragment of mRNA behaved differently, as dASO and rASO (200 nM) reduced EGFP-EGFR levels by 51% and 18%, respectively. This difference results from the distinct mechanisms of activity of these DNA and RNA antisense oligonucleotides. dASOs (DNA oligonucleotides) bind to their target mRNA oligomers of the sense sequence, and the resultant DNA/RNA heteroduplex activates the RNase H enzyme, which hydrolyses the mRNA strand and disrupts translation. In contrast, rASO (an RNA oligonucleotide) hybridizes to the target mRNA, excluding the mRNA from translation due to steric hindrance. Unfortunately, a single-stranded RNA activates neither RNase H nor an RNAi cascade, so the silencing effect of rASOs is rather limited. The Q-RNA segment, targeting G-quadruplex region of the mRNA did not affect the GFP-EGFR expression.

The silencing activity of two G-rich ASOs (QF-ASO and QL-ASO) was determined in the same DFA system. Results shown in Figure 5 demonstrate that both Q-ASOs exerted concentration-dependent silencing activities towards EGFR mRNA. Much such as dASOs, QF-ASOs reduced the expression of exogenous EGFR by 50%, while the oligomer QL-ASO, which hybridized more effectively *in vitro* with the target mRNA to form a G-quadruplex structure, reduced levels of exogenous EGFR mRNA by 70%. These results confirm that, besides the steric hindrance elicited by the rASO, an additional mechanism decreasing expression levels of the EGFP-EGFR fusion gene (likely intracellular formation of bimolecular G-quadruplexes) should be considered.

### 3.7. Mitochondrial Activity in HeLa Cells in Response to Transfection with dASO, rASO, Q-RNA, QF-ASO, and QL-ASO Antisense Oligonucleotides

**Figure 6:**
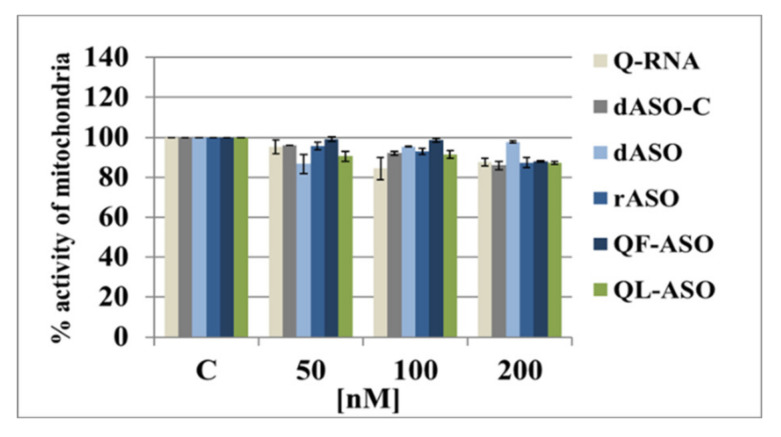
Cytotoxicity of tested ASOs, Q-RNA and Q-ASOs towards in HeLa cells as measured by MTT assay. Cells were transfected with oligomers at a concentration range of 50–200 nM using Lipofectamine and incubated for 48 h. Then, cell viability was assessed with the MTT assay as described in the Materials and Methods. Results are shown as the means ± standard deviation.

The viability of HeLa cells transfected with dASO, rASO, Q-RNA, QF-ASO, or QL-ASO antisense oligonucleotides, as well as with the dASO-C (control oligonucleotide), was measured using the widely known [3-(4,5-dimethylthiazol-2-yl)-2,5-diphenyltetrazolium bromide] (MTT) assay. HeLa cells were subjected to Lipofectamine-mediated transfection with the tested ASOs (50–200 nM), and mitochondrial activity was determined after 48 h of incubation. As shown in Figure 6, only a 10–20% decrease was observed, even if the oligomers were used at the highest (200 nM) concentrations. Low cytotoxicity observed in response to QF-ASO and QL-ASO is hypothetically beneficial for their therapeutic adaptation.

### 3.8. Analysis of Silencing Activity of QL-ASO Oligonucleotides by Microscopic Fluorescence Imaging

**Figure 7:**
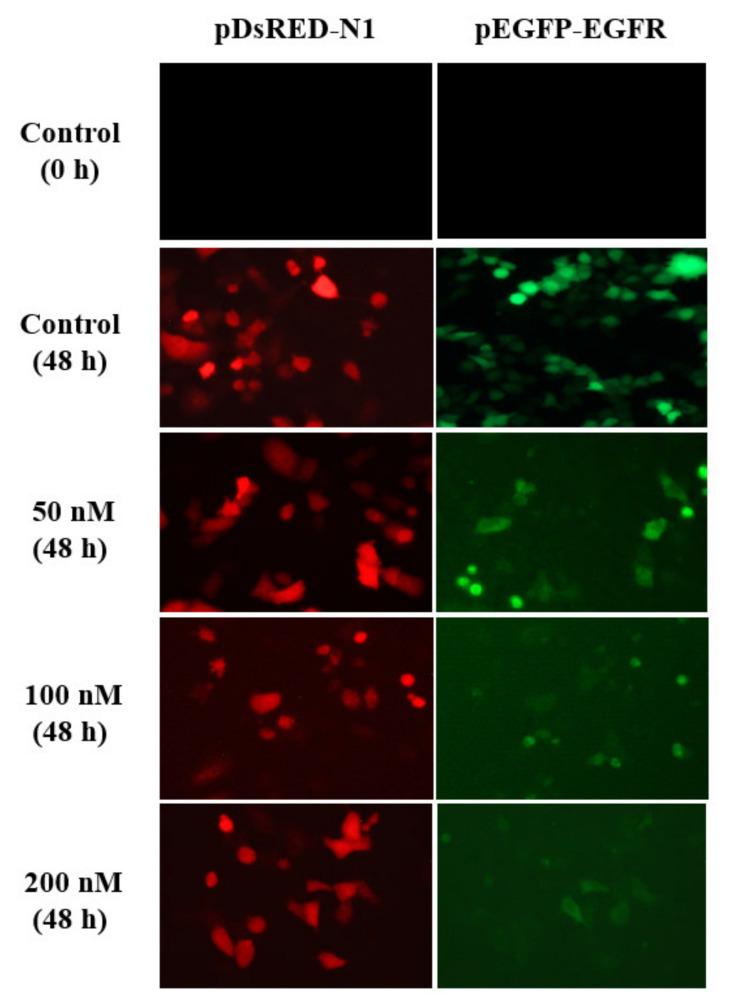
Silencing activity of modified oligonucleotide QL-ASO as monitored by fluorescence microscopy of EGFP-EGFR fusion protein expression in HeLa cells. Cells transfected with the pEGFP-EGFR and pDsRED-N1 plasmids with the dASO-C oligomer (100 nM) were used as controls (at time 0 and 48 h).

The silencing activity of the most active QL-ASO antisense oligonucleotide was monitored by imaging HeLa cells in the EGFP-EGFR/RFP double fluorescence model. In a control experiment, HeLa cells were subjected to Lipofectamine-mediated transfection with EGFP-EGFR/RFP plasmids and then with the control dASO-C oligomer (100 mM), while the remaining cells were transfected with antisense QL-ASO (50, 100, and 200 nM). Living cells were imagined using fluorescence microscopy measurements. As shown in Figure 7, the green fluorescence intensity of cells (see the p-EGFP-EGFR column) decreased with increasing concentrations of the test compound, while levels of red fluorescence intensity (pDsRED-N1 column) remained constant compared to control cells at 48 h. In the presence of a 200 nM concentration of QL-ASO, the density of the cells expressing EGFR was lower than that among the control cells and severely decreased cell proliferation was observed in response to decreasing EGF receptor availability.

### 3.9. Visualization of Density of the Endogenous EGFR mRNA Monitored by the G4-Detection in MCF-7, HeLa and A431 Cancer Cells

A characteristic feature of cancer cells is an increased number of epidermal growth factor receptors (EGFR) on the outer cell membranes and in cellular organelles. These receptors are important for maintaining uncontrolled growth of cancerous tumors and metastasis to other organs, and their quantity correlates well with the aggressiveness of cancer cells and their resistance to chemo- and radiotherapy. In our tests, MCF-7 (breast cancer), HeLa (cervical cancer), and A431 (epidermoid carcinoma) cancer cells with different densities of EGF receptors were used. Levels of the EGFR protein in MCF-7 cells were reportedly the lowest compared to HeLa and A431 cells [45,46]. The data collected from *in vitro* experiments demonstrated slower growth of MCF-7 tumor cells relative to HeLa cells [47,48]. In contrast, due to the high levels of EGFR, A431 cells are characterized by fast growth and proliferation, as well as high resistance to chemo- and radiotherapy [49,50]. Therefore, the epidermoid carcinoma exhibits the highest aggressiveness and the least optimistic therapeutic prognosis [50].

**Figure 8:**
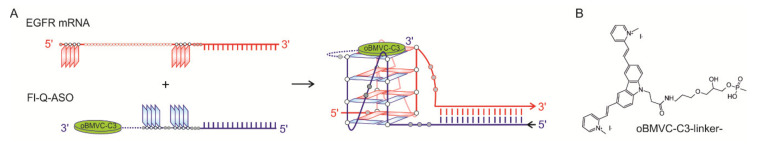
Schematic representation of the Fl-Q-ASO, EGFR mRNA and quadruplex-duplex hybrid containing a covalently attached ligand oBMVC-C3 (Fl-Q-ASO/EGFR) (**A**) and chemical structure of a oBMVC-C3 attached to 3′-end of Q-ASO-NH_2_ 3′-amino-oligonucleotide (**B**).

**Figure 9:**
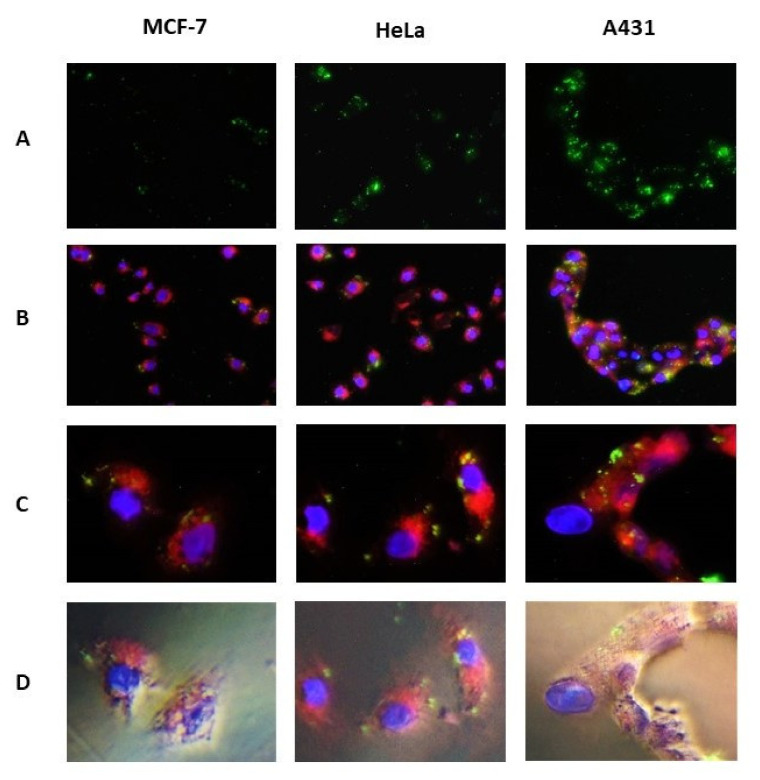
Microscopic analysis of localization of G-quadruplexes formed by Fl-Q-ASO (100 nM) and EGFR mRNA in MCF-7, HeLa and A431 cells. G4s are shown in green (indicated by the white arrows), cell ER membranes are stained in red (ER-Tracker Red), and dsDNA in the cell nuclei are stained in blue (DAPI). Panel **D**: phase contrast merged with the images shown in the panel **C**. Panels (**A**,**B**): 20-fold enlargement. Panels (**C**,**D**): 60-X magnification.

To determine whether levels of EGFR mRNA in the three tested cell lines correlated with the reported data, we decided to visualize the G-quadruplex domains of the hybrid structures using an anti-EGFR-mRNA oligonucleotide conjugated to a fluorescent probe Fl (Fl-Q-ASO). At the 3′-end, this Fl-Q-ASO contains 3,6-bis(1-methyl-4-vinylpyridinium) carbazole diiodide substituted with propionic acid at N9 (oBMVC-C3-linker-Q-ASO, Table 1, Figure S7), which in the Fl-Q-ASO/EGFR complex in vitro (Figure 8, Figure S8) or inside a cell in close proximity to a G-quadruplex becomes a green fluorophore (Figure 9). In free-floating Fl-Q-ASO, the probe remains inactive, and neither is seen in the cells nor in the cell medium. The complementary ASO fragment is expected to assure high sequence specificity and recognition of the adjacent G-tracts in the target EGFR mRNA. The G-rich specific antisense binding of the Fl-Q-ASO to C-rich sites of any mRNA would result in the absence of G4 fluorescent hybrids.

To determine the density of EGFR mRNA monitored by G4 detection, transfected cells (100 nM Fl-Q-ASO/Lipofectamine 2000, 5 h) were treated with ER-Tracker Red to stain the endoplasmic reticulum (ER) and with DAPI (to stain the dsDNA in the nucleus) and were examined by fluorescence microscopy. Formation of the Fl-Q-ASO/EGFR hybrid labeled with the oBMVC-C3 fluorescent probe was confirmed by non-denaturing PAGE (Figure S8) and by microscopic imaging (Figure 9). The signals appear in dot-like structures in many cells in cytoplasm/endoplasmic reticulum, the sites the EGFR mRNA takes part in protein synthesis. The least intense green fluorescence signal was observed in MCF-7 cells (ca. 1 green spot per cell), then for HeLa cells (ca. 3 green spots per cell), and the strongest was obtained in A431 cells (ca. 12 green spots per cell, Figure 9A). This result confirms the formation of quadruplex-duplex hybrid structures by the Q-ASO oligonucleotide conjugated to the oBMVC-C3-linker with the cytoplasmic fraction of EGFR mRNA (Figure 9; B and C are 20- and 60-X magnifications, respectively). Thus, the levels of endogenous EGFR mRNA templates detected here correlate well with the density of EGFR reported in these specific cell lines.

To confirm co-localization of Fl-Q-ASO and EGFR mRNA in tumor cells and to assess cell morphology, phase-contrast/fluorescence images were collected at various excitation wavelengths (blue fluorescence mode, λ_ex_ = is 358 nm, green fluorescence mode, λ_ex_ = 458/500 nm; red fluorescence mode, λ_ex_ = 587 nm) (Figure 9D). Interestingly, high levels of blue fluorescence and large nuclei, as well as a high intensity of red fluorescence, were induced in the ribosomal endoplasmic reticulum in A431 cells (Figure 9C), suggesting intense protein biosynthesis, which is characteristic for rapid cell proliferation in A431 cells and represents a highly aggressive skin squamous carcinoma.

### 3.10. Analysis of Hybridization of Fl-Q-ASO with Endogenous EGFR mRNA in A431 Cells in the Presence of Various Inhibitory ASOs

**Figure 10:**
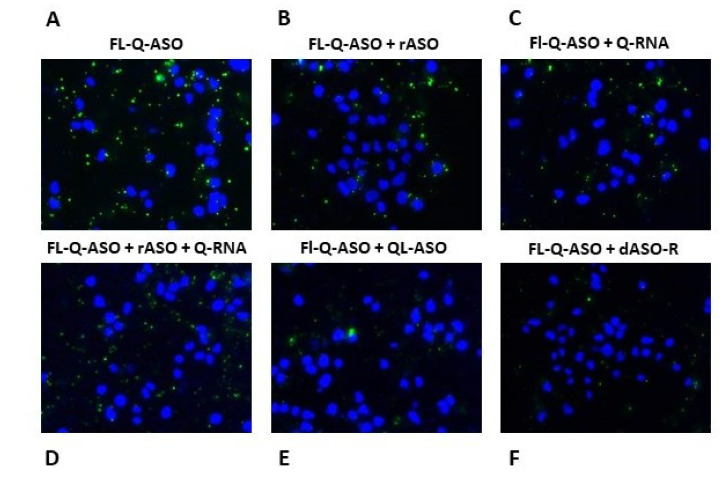
Fluorescence analysis of G4 green fluorescent structures in A431 cells, which were stained with DAPI (visualization of dsDNA in nuclei) and transfected with Fl-Q-ASO (100 nM) (**A**) along with the following inhibitory ASOs (100 nM): rASO (**B**), Q-RNA (**C**), rASO+QRNA (1:1 molar ratio) (**D**), QL-ASO (**E**) and dASO-R (**F**).

In line with our hypothesis, the oBMVC-C3-linker-Q-ASO binds strongly to EGFR mRNA and forms a hybrid structure comprising the bimolecular G-quadruplex, which was visualized by the fluorescent indicator present in close vicinity (Figure 9). The present experiment was intended to confirm the specificity of the association of Fl-Q-ASO to EGFR mRNA by altering the levels of mRNA and the process monitoring by G4-visualization. Thus, A431 cells were simultaneously transfected with Fl-Q-ASO (100 nM) in addition to oligonucleotides (100 nM), which were expected to compete with Fl-Q-ASO for the target mRNA by preventing the formation of Fl-Q-ASO/mRNA hybrids, reducing the green fluorescence signal. Initially, A431 cells were additionally transfected with rASO (an antisense control of Q-ASO) or Q-RNA (a G-rich region of Q-ASO) or with a 1:1 mixture of both, resulting in a substantial reduction in green fluorescence signal (Figure 10B–D) compared to cells transfected with Fl-Q-ASO only (Figure 10A). The addition of QL-ASO resulted in a 90% decrease of green fluorescence (Figure 10E), perhaps due to inhibition of the target mRNA by QL-ASO prior to its hybridization with Fl-Q-ASO. We assume that the Fl-Q-ASO oligonucleotide, due to the presence at its 3′-end the bulky amino-linker-bound oBMVC-C3 fluorescent tag, recognizes and hybridizes to the EGFR mRNA with slower kinetics than do the shorter DNA and RNA probes. Therefore, the accessibility of the target mRNA to Fl-Q-ASO is limited in the presence of competes. Continuing the analysis, we transfected A431 cells with the dASO-R oligonucleotide, which is a reference antisense oligonucleotide directed towards another fragment of the EGFR mRNA known to be an efficient inhibitor of expression of exogenous and endogenous EGFR in a DFA test and in A431 cells (these data will be published elsewhere), respectively. Resultant images (Figure 10F) showed a 10% G4-induced fluorescence only compared to the control, indicating that the target mRNA was degraded fast enough to escape binding to Fl-Q-ASO. This is a two-step degradation initiated by RNase H (activated by formation of a DNA/RNA duplex) and completed by hydrolysis of the damaged mRNA by cellular nucleases. Therefore, we conclude that the above described experiments unequivocally confirm the sequence-specific binding of Fl-Q-ASO to EGFR mRNA. If, however, Fl-Q-ASO were to bind to other, potentially available G-tracts (without the guidance provided by the antisense “guide” ASO segment), then the number of observed fluorescent spots should not depend in any way on the accessibility of EGFR mRNA being altered, and this was not a case. Moreover, transfection of A431 cells with Fl-Q-ASO and the control (non-active, non-complementary) dASO-C had no influence on the density of the green fluorescent G4 spots (data not shown).

In summary, we demonstrated that Fl-Q-ASO exerts highly sequence-specific hybridization to the target mRNA, and the formation of the quadruplex-duplex hybrids results in inactivation of mRNA and inhibition of protein synthesis, as shown by the DFA assay (Figure 5). Thus, the bifunctional RNA oligonucleotides used in this study, containing a “guide” antisense segment and G-rich tracts forming G-quadruplexes, offer a potentially useful new tool for therapeutic intervention.

## 4. Discussion

### 4.1. Formation of Quadruplex-Duplex Hybrid Structures

Recent studies have identified 80,307 stem-loop-containing G-quadruplex sequences (SLQS) within the human genome, including 23,742 in pre-mRNAs and 2429 in mature mRNAs [5]. These fragments have the potential to form in cell unimolecular quadruplex-duplex hybrid structures harboring duplex stems within a loop (ST-QDH). Formation of five different types of unimolecular DNA hybrid structures [3] and their high stability have been demonstrated *in vitro* [2,4,5,51]. Each type represents disparate connections between a duplex and a G-quadruplex and the diverse G-quadruplex cores (Figure S9). The duplex stem or multiple stems can be embedded across the various edges of a G-quadruplex core, in both coaxial and orthogonal manners [2,3]. Given the canonical structure of the duplexes and the parallel topology of RNA G-quadruplexes, RNA SLQS should fold into quadruplex-duplex hybrids with a duplex stem instead of one propeller loop, and these two domains should be connected by an adapter sequence of nucleotides (construct Figure S9, V). Unimolecular RNA quadruplex-duplex hybrids were identified in the structures of Sc1, Spinach and Mango aptamers identified by SELEX screens [52,53]. G-quadruplex cores and the architecture of their quadruplex-duplex junctions were found to be unique. In these structures, G-quadruplex domains were separated from the duplex stems by a GUAU-tetrad (for Sc1) and CGUU-tetrad (for Spinach) or an adaptor nucleotides instead of base pairs (for Mango) [52,53]. In these aptamers, noncanonical G-quadruplex cores were observed. For example, for Spinach aptamer, unusual antiparallel topology of the G-quadruplex domain was determined [54]. A special structural scaffold in these RNA hybrids might be essential for the function of these aptamers.

Bimolecular quadruplex-duplex hybrids may form when cells are transfected with G-rich oligonucleotides. Structures of bimolecular QDHs are poorly characterized and are limited to DNA:RNA hybrid model with hetero-quadruplex domains adopting a parallel topology and a duplex stem mimicking an external loop [23,24]. This model can be described as belonging to construct V of unimolecular DNA QDHs. Our results provide the first data on bimolecular RNA:RNA hybrids. The RNA Q-ASO/EGFR hybrid is composed of a four-plane parallel quadruplex connected with a 16-bp duplex by three unpaired nucleotides on both strands (Figure 1). This model can be classified as construct V. Very recently, Phan et al. demonstrated that long structured loops can increase G4 folding [38]. Based on this study, we postulated that the folding process of Q-ASO and its EGFR target with distant G-tracts would be accelerated when the formation of the G-quadruplex core goes hand-in-hand with the formation of the duplex stems (2ST) (Figure 1). Of note, the bimolecular DNA/RNA and RNA:RNA hybrid structures preserve the same construction where parallel G-quadruplex and duplex helices are connected with no base stacking between the two components. The use of RNA Q-ASOs allowed us to obtain more stable RNA:RNA than DNA:RNA hybrid structures. The chemical modifications can also influence the stability of RNA hybrids. 2′-FANA-G (F) was primarily used to enforce the *anti* conformation of G residue which is the preferred conformation of parallel RNA G-quadruplexes. The presence of two GFFG-tracts in QF-ASO led to the formation of the QF-ASO/EGFR hybrid with the parallel G-quadruplex domain. In this G-quadruplex the two inner layers are composed of FFGG-tetrads. The second hybrid (QL-ASO/EGFR) comprises an unmodified G-core with abasic modifications and aliphatic linkers located in the junction region and in the external loop (Figure 4). It appeared that while maintaining the same hybrid structure, the presence of non-nucleotide modifications contributed more to the stability of the hybrid structure than 2′-FANA-G modification. Thus, an approach for designing RNA G-rich antisense oligonucleotides for the downregulation of G-tracts containing genes in a G4-mediated manner is proposed here.

### 4.2. The RNA Q-ASO Approach

ASO:mRNA hybridization relies upon the kinetic and thermodynamic parameters of RNA folding, oligonucleotide affinity, and association/dissociation rates [55]. The most frequently used method to enhance the efficiency of gene expression inhibition using the antisense approach is the introduction of different chemical modifications [56,57]. Modifications of the ribose are a common class of alterations. The most often used chemical modifications in this group are 2′-O-methyl, LNA (locked nucleic acid), 2′-O-methoxyethyl, 2′-fluoro, 2′-FANA (2′-deoxy-2′-fluoro-β-D-arabinonucleic acid) or (S)-cET (2′,4′-constrained ethyl nucleic acid) [58,59,60,61]. These modifications increase stability toward digestion by nucleases and also increase the strength of hybridization. For DNA ASO, in addition to induction of RNase H endonuclease activity that cleaves RNA within the DNA-mRNA heteroduplexes, other ASO-driven mechanisms were considered, including short G-rich molecules or guanine-tethered antisense strategies relying on formation of G-quadruplex or quadruplex-duplex hybrid structures on the mRNA template, respectively [23,24].

Here, we revealed a new, unique approach for the downregulation of EGFR via the dual-motif targeting of this gene using G-rich antisense RNA oligonucleotides (Q-ASO). A targeted sequence contains two important fragments, one complementary to a region of the Q-ASO oligonucleotide and another comprising two four-guanosine tracts separated by 28 nucleotides. After hybridization with Q-ASO, formation of quadruplex-duplex hybrid structures was confirmed in vitro and in living cells. We demonstrated that the use of RNA Q-ASO led to formation of bimolecular RNA-RNA G4 structures on mRNA templates with enhanced gene silencing ability compared to rASO and dASO. In this work, we introduced chemical modification in the G-quadruplex segment only and not duplex. The comparison of unmodified control antisense oligonucleotides (dASO, rASO) and Q-ASO modified in the G-quadruplex segment allows to better assess the impact of the selected modifications on the functionality of the designed bifunctional antisense tool. We indicated the advantage of using non-nucleotide modifications, especially such as abasic analogs between Q and ASO sequences or aliphatic linker between G-tracts. We postulate that the more flexible -aaa- chain and the longer linker S6 between G-tracts allows the QL-ASO for easier spatial accommodation of the G-strands to form the G-quadruplex in comparison to QF-ASO, containing corresponding -AAA- and -UC- linkers, instead (Figure 4). These modifications contributed to the thermodynamic stabilization of RNA G4-domains of 2ST-DQH hybrids which are an effective steric hindrance for ribosome, resulting in inhibition of protein synthesis. The formation of a QDH on the mRNA template appeared to be an effective steric hindrance for ribosome stalling and inhibiting protein synthesis. Our present studies aimed to obtain the proof-of-concept data on the formation of bimolecular RNA-RNA G4 structures on mRNA templates in vitro and in the cell. For therapeutic use one may consider the use of 2′-*O*-modified analogs of natural backbones to increase metabolic stability of RNA ASOs [62,63].

The identification of RNA G-quadruplex formation in living cells remains a challenge, especially in mRNAs. It was postulated by Guo and Bartel that RNA G4 can be globally unfolded in humans [64]. However, the relatively high DMS concentration (not single-hit kinetics conditions) used to chemical probing the RNA could influence the final result of the experiments or this method samples the unfolded state during the time course of the reaction [65]. The emerging evidence suggests that RNA G4 may be dynamic and fold only transiently between G-quadruplex and single-stranded forms *in vivo*, depending on cellular conditions, the presence of binding proteins, helicases, stress conditions, or the cell cycle phase. Recently, the use of the fluorescent probe QUMA-1 for the real-time visualization of endogenous RNA G-quadruplexes in living cells has been demonstrated [66]. To selectively visualize the endogenous G-quadruplexes formed by particular sequences (e.g., the *NRAS* mRNA 5′-UTR), use of a bifunctional fluorogenic probe, ISCH-nras1, was presented [67]. This probe is composed of a G4 fluorescent moiety and a DNA segment that can hybridize only with an *on*-target sequence [67,68]. In the present study, we covalently attached a well-studied fluorescent tag [69], i.e., oBMVC, to the 3′-end of Q-ASO. Microscopic analysis of the localization of G4 green fluorescent spots originating from oBMVC-C3-Q-ASO/EGFR hybrid structures confirmed the highly specific recognition of the target and the formation of quadruplex-duplex hybrid structures at a programmable site in living cells.

## 5. Conclusions

Herein, we propose an approach for downregulation of genes containing two distant G-tracts in a G4-mediated manner using RNA G-rich antisense oligonucleotides. We have provided evidence that the chemically modified RNA Q-ASO binds to the 56-nt EGFR mRNA fragment, resulting in the assembly of a bimolecular quadruplex-duplex hybrid structure (2ST-QDH). The formation of the stable G-quadruplex on mRNA template appeared to be an effective steric hindrance for ribosome and resulted in stalling and inhibition of protein synthesis. The silencing activity of QL-ASO G-rich antisense oligonucleotides toward the disease-associated EGFR mRNA target was at least 20% higher than for unmodified RNA or DNA antisense oligonucleotides. Finally, Q-ASO with covalently attached carbazole derivative oBMVC-C3 was used as an internal fluorescent probe. Sequence-specific binding of Q-ASO to the target mRNA was monitored in living cells by the observation of a green fluorescence of oBMVC-C3 moiety. The possibility to control formation of G-quadruplex by the use of G4-ligand linked to oligonucleotides can provide a basis for new investigations in the field of biology.

## Figures and Tables

**Table 1:** Sequences of RNA and DNA oligonucleotides (5′→3′) used in this study.

Reference Antisense Oligonucleotides	Name
d(AGCAGCGCCAGGAGCG)	dASO
r(AGCAGCGCCAGGAGCG)	rASO
r(AGGGGUCGGGGA)	Q-RNA
d(TTTCTTTTCCTCCAGAGCCCGA)	dASO-R
d(ATGAAGGTTCAATCTGATTTT)	dASO-C
**Q-ASO: G-rich antisense oligoribonucleotides folded into the quadruplex-duplex hybrid**	
AGCAGCGCCAGGAGCG-AAA-GFFG-UC-GFFGA	QF-ASO
AGCAGCGCCAGGAGCG-aaa-GGGG-S6-GGGGA	QL-ASO
AGCAGCGCCAGGAGCG-AAA-GGGG-UC-GGGGA-linker-NH_2_	Q-ASO-NH_2_
AGCAGCGCCAGGAGCG-AAA-GGGG-UC-GGGGA-linker-oBMVCC3	Fl-Q-ASO
**EGFR: EGFR-235-290 mRNA target sequence**	
C**GGGG**-*AGCAGCGAUGCGACCCUCCGGGACGGCC-***GGGG**-CAG- CGCUCCUGGCGCUGCU	EGFR

F: 2′-deoxy-2′-fluoro-D-arabinoguanosine (2′-FANA-G); a: abasic; S6: (CH_2_)_6_, 28-nt fragment of mRNA is indicated in italics.

## Supplementary Materials

The following are available online at https://www.mdpi.com/2073-4409/9/11/2375/s1. Figure S1. The 56-nucleotide fragment sequence of the EGFR target used in biophysical studies is marked with a red box. Figure S2. Putative model of bimolecular G-quadruplex of QF-ASO and QL-ASO. Figure S3. Secondary structures of the EGFR target (A), rASO/EGFR (B), QF-ASO/EGFR (C), QL-ASO/EGFR (D) as predicted by RNAstructure and quadruplex-duplex hybrid structure containing a two duplex stems at the external loop (2ST-QDH) (E); (GGGG-tract- red or navy blue circles, FG –purple circles, a or S6- white circles, any nucleotides- grey circle, base pairs: A:U—grey line, G:C—blue line, G:U—red line). Figure S4. Analysis of QF-ASO, EGFR, QF-ASO/EGFR and QL-ASO/EGFR structures by non-denaturing PAGE. All oligomers in 0.1 M Tris HCl, pH 6.8, with 50 mM KCl, 150 mM LiCl and 2 equivalents of G4-ligand: NMM was annealed and subjected to non-denaturing PAGE. Next, the gel was visualized by UV shadowing (A) and exposed to 532 nm light (B). Figure S5. Imino region of the 1H NMR spectra of 28-nt molecule (AGCAGCGAUGCGACCCUCCG-GGACGGCC) corresponding to mRNA fragment of sequence located between two G-tracts in EGFR target. Spectra were recorded at 25 °C and 40 °C in 90% H2O/10% D2O (*v/v*) in the presence of 50 mM KCl, 150 mM LiCl, 10 mM Tris-HCl, 0.1 mM EDTA, pH 6.8. Figure S6. CD spectra of QF-ASO/EGFR and QL-ASO/EGFR hybrids at 25 °C in potassium buffer. Figure S7. oBMVC-C3 labeling of Q-ASO-NH2 oligonucleotides. Figure S8. As a control, we used non-covalent complex of QL-ASO/EGFR hybrid with NMM. After electrophoresis, the gel was exposed to 532 nm light to detect bands corresponding to the Fl-Q-ASO/EGFR and NMM:QL-ASO/EGFR hybrids. QL-ASO/EGFR in NMM, 50 mM KCl, 150 mM LiCl, 100 mM Tris HCl, pH 6.8 and Fl-Q-ASO/EGFR in 50 mM KCl and 150 mM LiCl, 100 mM Tris HCl, pH 6.8 were annealed and subjected to non-denaturing PAGE. Figure S9. Different type of the unimolecular quadruplex–duplex hybrids. Figure S10. (A) RP-HPLC profile of the purified dASO (5′-d(AGC AGC GCC AGG AGC G)-3′) after automatic synthesis; (B) Q-TOF MS: MW calc. (g/mol): 4941.22; m/z 4941.8203. Figure S11. (A) RP-HPLC profile of the purified rASO (5′-AGC AGC GCC AGG AGC G-3′) after automatic synthesis; (B) Q-TOF MS: MW calc. (g/mol): 5197.21; m/z 5197.7002. Figure S12. (A) RP-HPLC profile of the purified oligonucleotide 5′-AGG GGU CGG GGA-3′ (Q-RNA) after automatic solid phase synthesis; (B) Q-TOF MS: MW calc. (g/mol): 3969.44, m/z 3968.6001. Figure S13. The purity and homogeneity of Q-ASO, Fl-Q-ASO and EGFR mRNA oligomers verified by 15% denaturing gel electrophoresis.
