# Supplementary material for: Formation of an RNA Quadruplex-Duplex Hybrid in Living Cells between mRNA of the Epidermal Growth Factor Receptor (EGFR) and a G-Rich Antisense Oligoribonucleotide
Source: Cells. 2020 Oct 29;9(11):2375. doi: 10.3390/cells9112375 (PMC7692301; doi:10.3390/cells9112375)
Supplement: Supplementary file 1 [file cells-09-02375-s001.pdf]

Formation of an RNA Quadruplex-Duplex Hybrid in Living Cells Between mRNA of the Epidermal Growth Factor Receptor (EGFR) and a G-Rich Antisense Oligoribonucleotide

Dorota Gudanis[1,&,*], Damian Kaniowski[2,&], Katarzyna Kulik[2], Daniel Baranowski[1], Zofia Gdaniec[1], Barbara Nawrot[2]

[1] Department of Biomolecular NMR, Institute of Bioorganic Chemistry, Polish Academy of Sciences, Poznan, 61-704, Poland

[2] Centre of Molecular and Macromolecular Studies, Polish Academy of Sciences, Lodz, 90-363, Poland;

&- equal first authorship

* corresponding author
Email: dgud@ibch.poznan.pl

Supplementary Information

Figure S1. The 56-nucleotide fragment sequence of the EGFR target used in biophysical studies is marked with a red box.

Figure S2. Putative model of bimolecular G-quadruplex of QF-ASO and QL-ASO.

Figure S3. Secondary structures of the EGFR target (A), rASO/EGFR (B), QF-ASO/EGFR (C), QL-ASO/EGFR (D) as predicted by RNAstructure and quadruplex-duplex hybrid structure containing a two duplex stems at the external loop (2ST-QDH) (E); (GGGG-tract- red or navy blue circles, FG –purple circles, a or S6- white circles, any nucleotides- grey circle, base pairs: A:U – grey line, G:C – blue line, G:U – red line).

Figure S4. Analysis of QF-ASO, EGFR, QF-ASO/EGFR and QL-ASO/EGFR structures by non-denaturing PAGE. All oligomers in 0.1 M Tris HCl, pH 6.8, with 50 mM KCl, 150 mM LiCl and 2 equivalents of G4-ligand: NMM was annealed and subjected to non-denaturing PAGE. Next, the gel was visualized by UV shadowing (A) and exposed to 532 nm light (B).

Figure S5. Imino region of the 1H NMR spectra of 28-nt molecule (AGCAGCGAUGCGACCCUCCG-GGACGGCC) corresponding to mRNA fragment of sequence located between two G-tracts in EGFR target. Spectra were recorded at 25 °C and 40 °C in 90% H2O / 10 % D2O (v/v) in the presence of 50 mM KCl, 150 mM LiCl, 10 mM Tris-HCl, 0.1 mM EDTA, pH 6.8.

Figure S6. CD spectra of QF-ASO/EGFR and QL-ASO/EGFR hybrids at 25 °C in potassium buffer.

Figure S7. oBMVC-C3 labeling of Q-ASO-NH2 oligonucleotides.

Figure S8. As a control, we used non-covalent complex of QL-ASO/EGFR hybrid with NMM. After electrophoresis, the gel was exposed to 532 nm light to detect bands corresponding to the Fl-Q-ASO/EGFR and NMM:QL-ASO/EGFR hybrids. QL-ASO/EGFR in NMM, 50 mM KCl, 150 mM LiCl,

100 mM Tris HCl, pH 6.8 and Fl-Q-ASO/EGFR in 50 mM KCl and 150 mM LiCl, 100 mM Tris HCl, pH 6.8 were annealed and subjected to non-denaturing PAGE.

Figure S9. Different type of the unimolecular quadruplex–duplex hybrids.

Figure S10. (A) RP-HPLC profile of the purified dASO (5'-d(AGC AGC GCC AGG AGC G)-3') after automatic synthesis; (B) Q-TOF MS: MW calc. (g/mol): 4941.22; m/z 4941.8203.

Figure S11. (A) RP-HPLC profile of the purified rASO (5’-AGC AGC GCC AGG AGC G-3’) after automatic synthesis; (B) Q-TOF MS: MW calc. (g/mol): 5197.21; m/z 5197.7002.

Figure S12. (A) RP-HPLC profile of the purified oligonucleotide 5’-AGG GGU CGG GGA-3’ (Q-RNA) after automatic solid phase synthesis; (B) Q-TOF MS: MW calc. (g/mol): 3969.44, m/z 3968.6001.

Figure S13. The purity and homogeneity of Q-ASO, Fl-Q-ASO and EGFR mRNA oligomers verified by 15% denaturing gel electrophoresis.

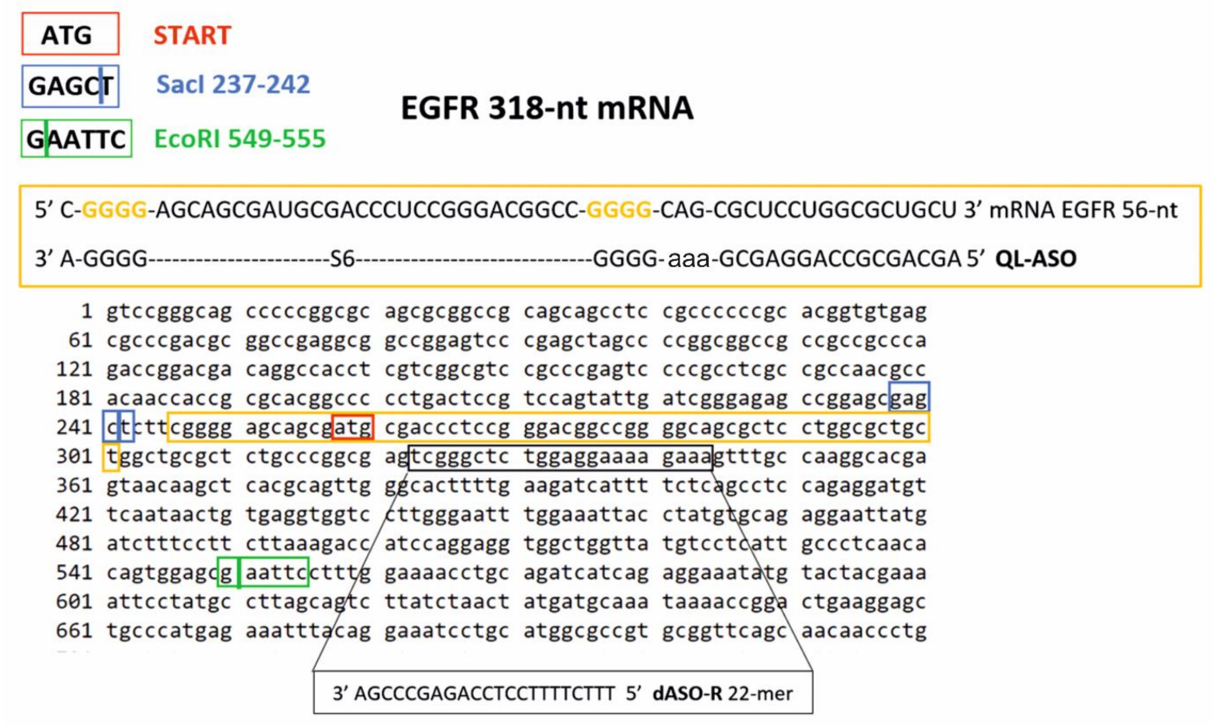

Figure S1. The 56-nucleotide fragment sequence of the EGFR target used in biophysical studies is marked with a red box.

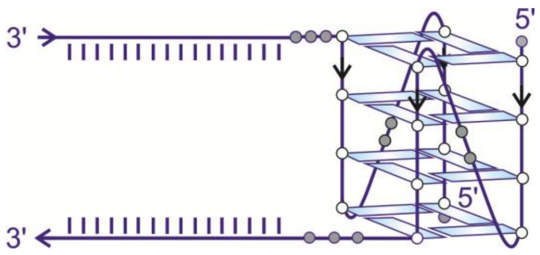

Figure S2. Putative model of bimolecular G-quadruplex of QF-ASO and QL-ASO.

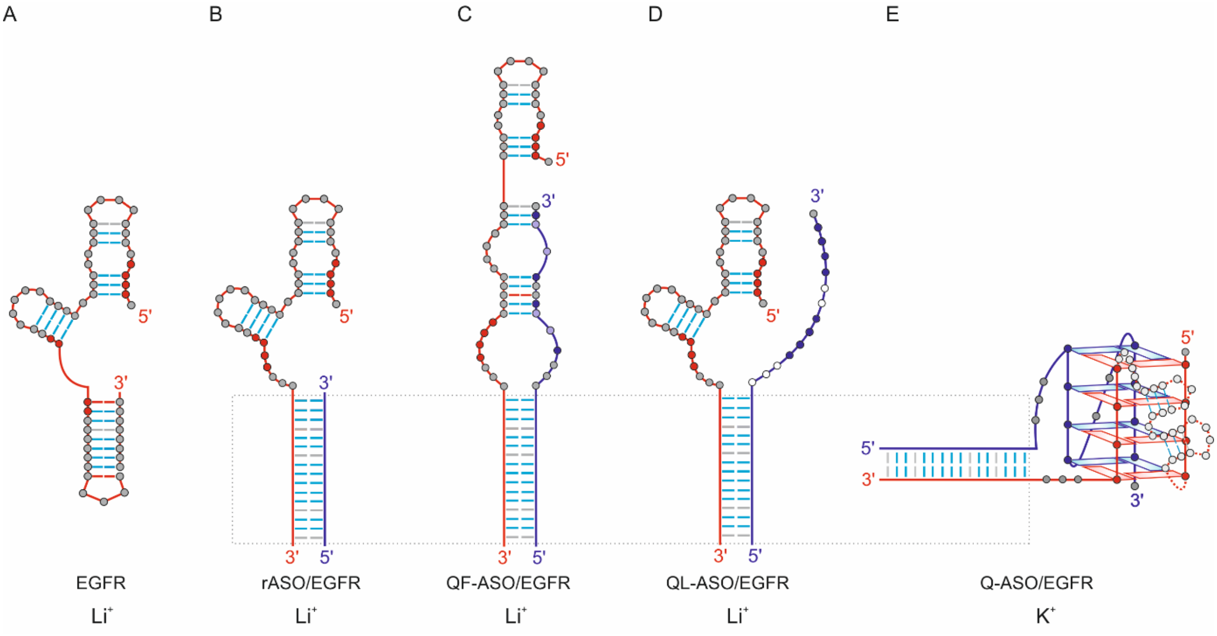

Figure S3. Secondary structures of the EGFR target (A), rASO/EGFR (B), QF-ASO/EGFR (C), QL-ASO/EGFR (D) as predicted by RNAstructure and quadruplex-duplex hybrid structure containing a two duplex stems at the external loop (2ST-QDH) (E); (GGGG-tract- red or navy blue circles, FG –purple circles, a or S6- white circles, any nucleotides- grey circle, base pairs: A:U – grey line, G:C – blue line, G:U – red line).

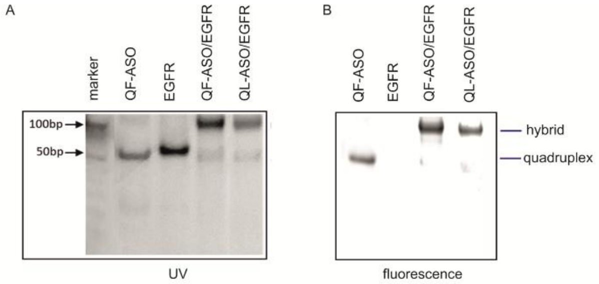

Figure S4. Analysis of QF-ASO, EGFR, QF-ASO/EGFR and QL-ASO/EGFR structures by non-denaturing PAGE. All oligomers in 0.1 M Tris HCl, pH 6.8, with 50 mM KCl, 150 mM LiCl and 2 equivalents of G4-ligand: NMM was annealed and subjected to non-denaturing PAGE. Next, the gel was visualized by UV shadowing (A) and exposed to 532 nm light (B).

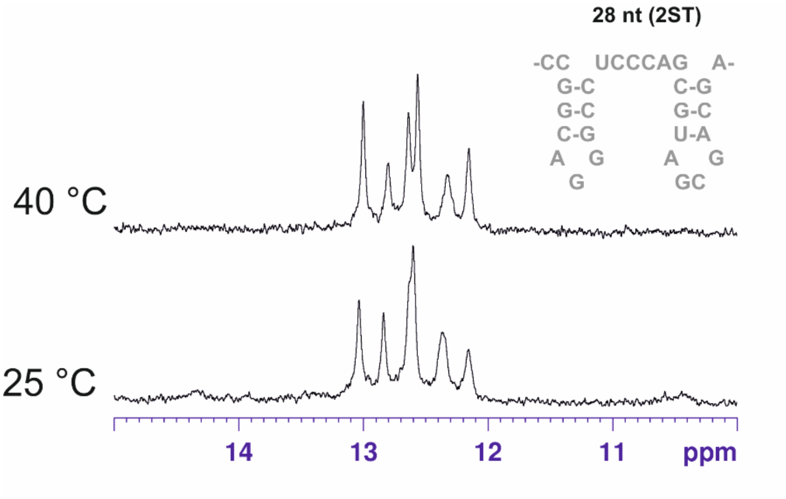

Figure S5. Imino region of the 1H NMR spectra of 28-nt molecule (AGCAGCGAUGCGACCCUCCG-GGACGGCC) corresponding to mRNA fragment of sequence located between two G-tracts in EGFR target. Spectra were recorded at 25 °C and 40 °C in 90% H2O / 10 % D2O (v/v) in the presence of 50 mM KCl, 150 mM LiCl, 10 mM Tris-HCl, 0.1 mM EDTA, pH 6.8.

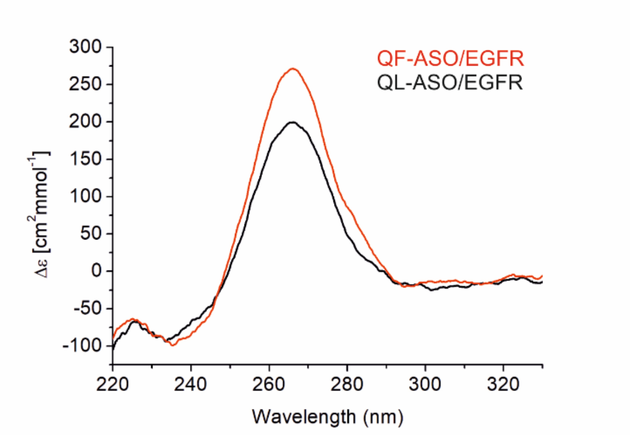

Figure S6. CD spectra of QF-ASO/EGFR and QL-ASO/EGFR hybrids at 25 °C in potassium buffer.

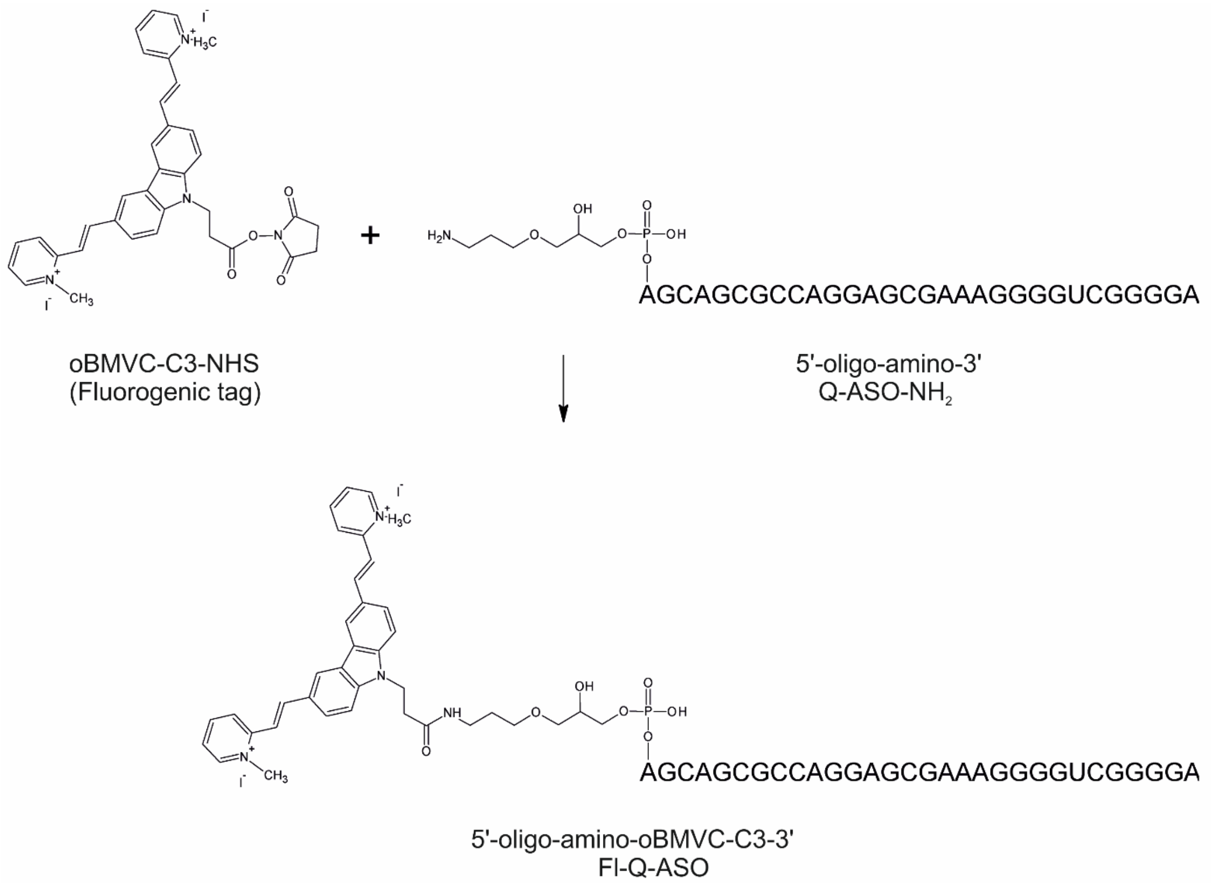

Figure S7. oBMVC-C3 labeling of Q-ASO-NH2 oligonucleotides.

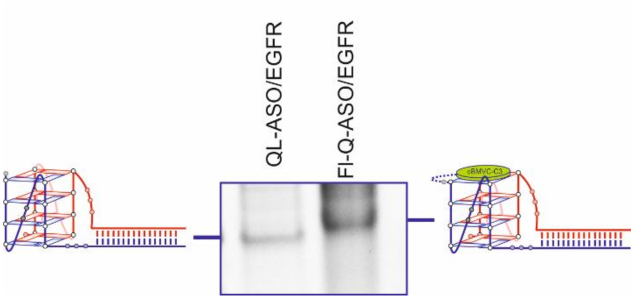

Figure S8. As a control, we used non-covalent complex of QL-ASO/EGFR hybrid with NMM. After electrophoresis, the gel was exposed to 532 nm light to detect bands corresponding to the Fl-Q-ASO/EGFR and NMM:QL-ASO/EGFR hybrids. QL-ASO/EGFR in NMM, 50 mM KCl, 150 mM LiCl, 100 mM Tris HCl, pH 6.8 and Fl-Q-ASO/EGFR in 50 mM KCl and 150 mM LiCl, 100 mM Tris HCl, pH 6.8 were annealed and subjected to non-denaturing PAGE.

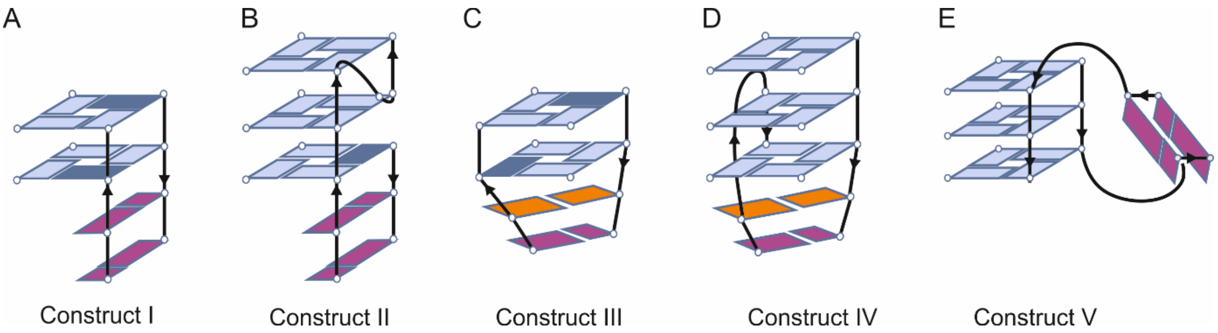

Figure S9. Different type of the unimolecular quadruplex–duplex hybrids.

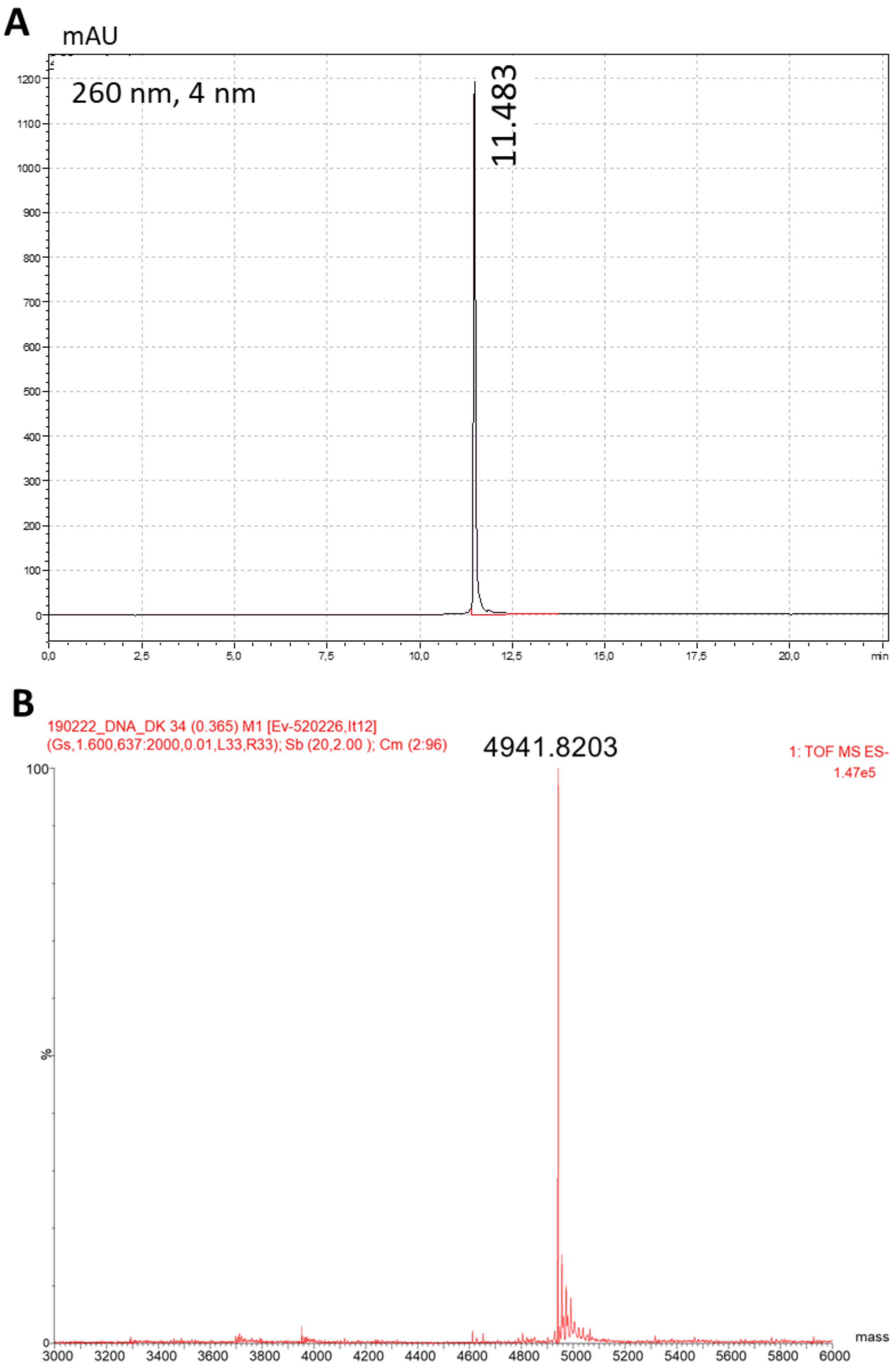

Figure S10. (A) RP-HPLC profile of the purified dASO (5'-d(AGC AGC GCC AGG AGC G)-3') after automatic synthesis; (B) Q-TOF MS: MW calc. (g/mol): 4941.22; m/z 4941.8203.

A

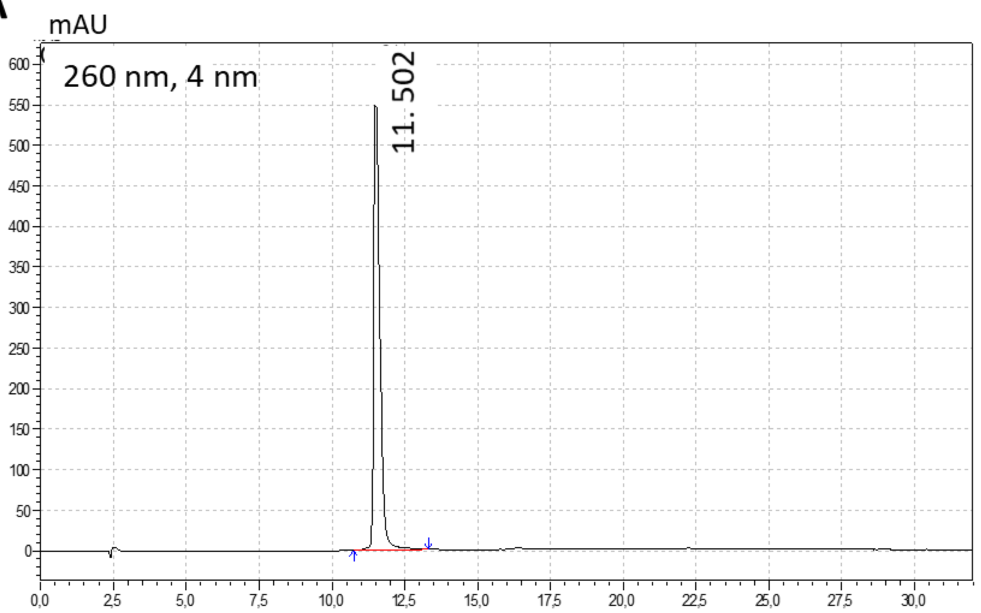

B

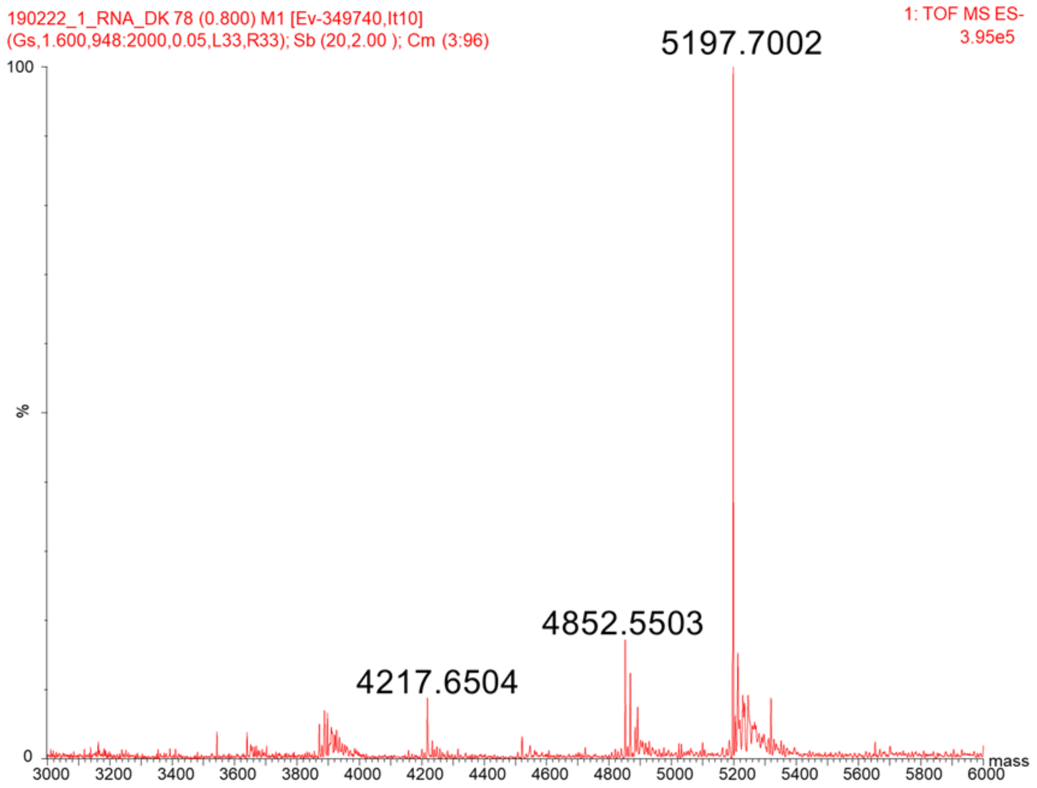

Figure S11. (A) RP-HPLC profile of the purified rASO (5'-AGC AGC GCC AGG AGC G-3') after automatic synthesis; (B) Q-TOF MS: MW calc. (g/mol): 5197.21; m/z 5197.7002.

A

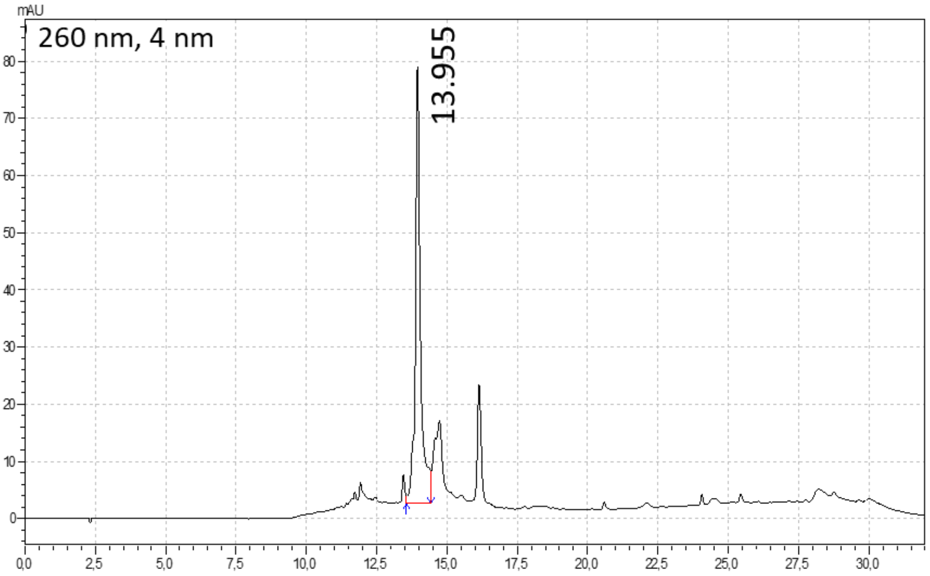

B

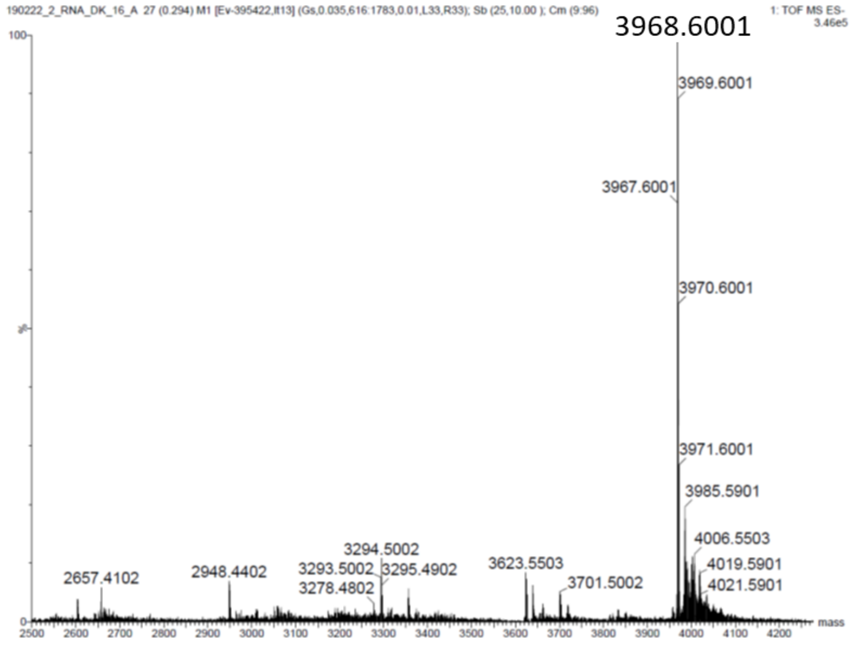

Figure S12. (A) RP-HPLC profile of the purified oligonucleotide 5'-AGG GGU CGG GGA-3' (Q-RNA) after automatic solid phase synthesis; (B) Q-TOF MS: MW calc. (g/mol): 3969.44, m/z 3968.6001.

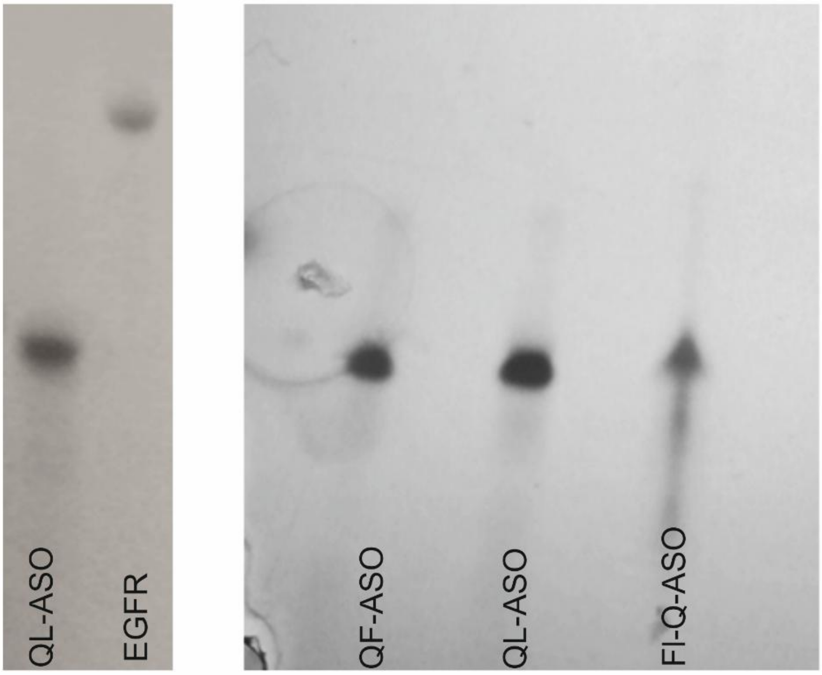

Figure S13. The purity and homogeneity of Q-ASO, Fl-Q-ASO and EGFR mRNA oligomers verified by 15% denaturing gel electrophoresis.
